# Large-scale production of megakaryocytes from human pluripotent stem cells by chemically defined forward programming

**DOI:** 10.1038/ncomms11208

**Published:** 2016-04-07

**Authors:** Thomas Moreau, Amanda L. Evans, Louella Vasquez, Marloes R. Tijssen, Ying Yan, Matthew W. Trotter, Daniel Howard, Maria Colzani, Meera Arumugam, Wing Han Wu, Amanda Dalby, Riina Lampela, Guenaelle Bouet, Catherine M. Hobbs, Dean C. Pask, Holly Payne, Tatyana Ponomaryov, Alexander Brill, Nicole Soranzo, Willem H. Ouwehand, Roger A. Pedersen, Cedric Ghevaert

**Affiliations:** 1 Department of Haematology, University of Cambridge and NHS Blood and Transplant, Long Road, Cambridge CB2 0PT, UK; 2 The Anne McLaren Laboratory, Wellcome Trust-Medical Research Council Cambridge Stem Cell Institute and Department of Surgery, University of Cambridge, West Forvie Site, Robinson Way, Cambridge CB2 0SZ, UK; 3 Wellcome Trust-Medical Research Council Cambridge Stem Cell Institute, Tennis Court Road, Cambridge CB2 1QR, UK; 4 Human Genetics, Wellcome Trust Sanger Institute, Genome Campus, Hinxton CB10 1RQ, UK; 5 Institute of Cardiovascular Sciences, University of Birmingham, Edgbaston, Birmingham B15 2TT, UK

## Abstract

The production of megakaryocytes (MKs)—the precursors of blood platelets—from human pluripotent stem cells (hPSCs) offers exciting clinical opportunities for transfusion medicine. Here we describe an original approach for the large-scale generation of MKs in chemically defined conditions using a forward programming strategy relying on the concurrent exogenous expression of three transcription factors: GATA1, FLI1 and TAL1. The forward programmed MKs proliferate and differentiate in culture for several months with MK purity over 90% reaching up to 2 × 10^5^ mature MKs per input hPSC. Functional platelets are generated throughout the culture allowing the prospective collection of several transfusion units from as few as 1 million starting hPSCs. The high cell purity and yield achieved by MK forward programming, combined with efficient cryopreservation and good manufacturing practice (GMP)-compatible culture, make this approach eminently suitable to both *in vitro* production of platelets for transfusion and basic research in MK and platelet biology.

## Abstract (web summary)

Platelets are blood circulating corpuscles generated from megakaryocytes that initiate wound healing. Here, Moreau *et al*. describe a way of producing large quantities of megakaryocytes from human pluripotent stem cells in the laboratory, moving us a step closer to manufacturing transfusion products.

## Graphical abstract

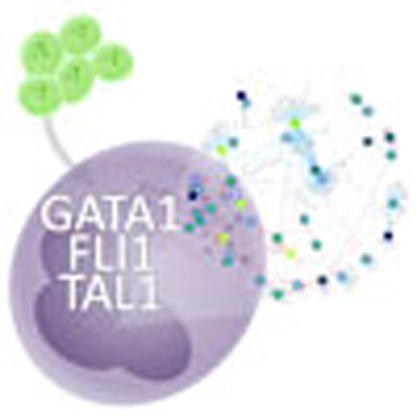

Megakaryocytes (MKs) generate blood platelets whose primary role is to stop haemorrhages via localized clot formation at the site of vessel injury^1^,^2^. MKs are polyploid cells derived from haematopoietic stem cells residing in the bone marrow where they represent only 0.01% of the total nucleated blood cells. By extension of cytoplasmic protrusions through bone marrow sinusoids, they release daily ∼1 × 10^11^ platelets into the blood stream to sustain the count of short-lived (7–10 days) circulating platelets between 150–450 × 10^9^ per litre of blood^3^,^4^. A decrease in platelet number, or thrombocytopenia, may occur following bone marrow failure (inherited or acquired, such as post-cancer treatment) or severe peripheral bleeding after trauma or surgery, and potentially leads to life-threatening haemorrhages. Currently, prophylactic and therapeutic treatment essentially relies on transfusion of ABO and Rhesus-D-matched platelet concentrates—at >2.4 × 10^11^ platelets per unit—from voluntary donations^5^,^6^. Recently, the increase in high-dose cancer therapy, advanced surgical procedures and the ageing population has led to a rising demand for platelets with over 4.5 million platelet units transfused per year in Europe and the United States^7^. In addition, platelet transfusion refractoriness in HLA class I alloimmunized chronically transfused patients and multiparous women necessitates the special provision of matched platelet units sourced from a small pool of genotyped recallable donors^8^. Altogether, the dependence on donations combined with the limited shelf life of platelet concentrates (5–7 days) represents a logistical, financial and biosafety challenge for health organizations worldwide.

Human pluripotent stem cells (hPSCs)—including embryonic stem cells (hESCs) derived from embryos and induced PSCs (hiPSCs) generated from post-natal somatic cells—can be maintained *in vitro* for prolonged periods while retaining the capacity to differentiate towards virtually any cell type upon adequate stimulation^9^,^10^,^11^. Therefore, they offer huge opportunities for basic research and clinical applications^12^. The production of platelets *in vitro* from genetically defined hPSC lines could revolutionize transfusion medicine by providing a controllable source of platelets^13^. Moreover, platelets are anucleate and do not proliferate which means they can be irradiated before transfusion. This provides a marked safety advantage over other hPSC-derived therapeutic cells which can potentially retain oncogenic cell fractions^14^. However, *in vitro* systems for the production of large amounts of MKs and subsequent platelet release to match the needs for making transfusion units still require considerable optimization.

Our work describes a novel approach for generating large quantities of functional MKs from hPSCs with unique advantages for clinical development. Existing protocols have so far relied on external signals provided by cytokines or stromal cells to mimic embryonic development *in vitro* and thus direct sequential differentiation of hPSCs into MKs, a process designated as ‘directed differentiation’^15^,^16^,^17^,^18^,^19^,^20^. While mature MKs showing functional platelet release are produced, this strategy has been limited by the relatively low number of MKs generated or by the complex genetic modifications and clonal selection required to immortalize MKs post differentiation. Urged by the recent discoveries on the plasticity of cell identities controlled by limited sets of transcription factors (TFs)^21^, we adopted a radically different approach for the generation of MKs by exploring the potential of exogenous TFs to drive the differentiation process from hPSCs, a strategy called ‘forward programming’ (FOP). Proceeding from a methodically curated list of candidate genes, we discovered that the combination of GATA1–FLI1–TAL1 uniquely promoted highly efficient MK-FOP from an array of hPSC lines in chemically defined conditions. Critically, the forward programmed MKs (fopMKs) matured into platelet-producing cells that could be cryopreserved, maintained and amplified *in vitro* for over 90 days showing an average yield of 200,000 MKs per input hPSC. This unprecedented efficiency combined with minimal cell manipulation and low cytokine requirements makes MK-FOP a promising platform for basic research as well as future clinical applications in the field of transfusion medicine.

## Results

### GATA1–FLI1–TAL1 induces hPSC to MK commitment

**Figure 1:**
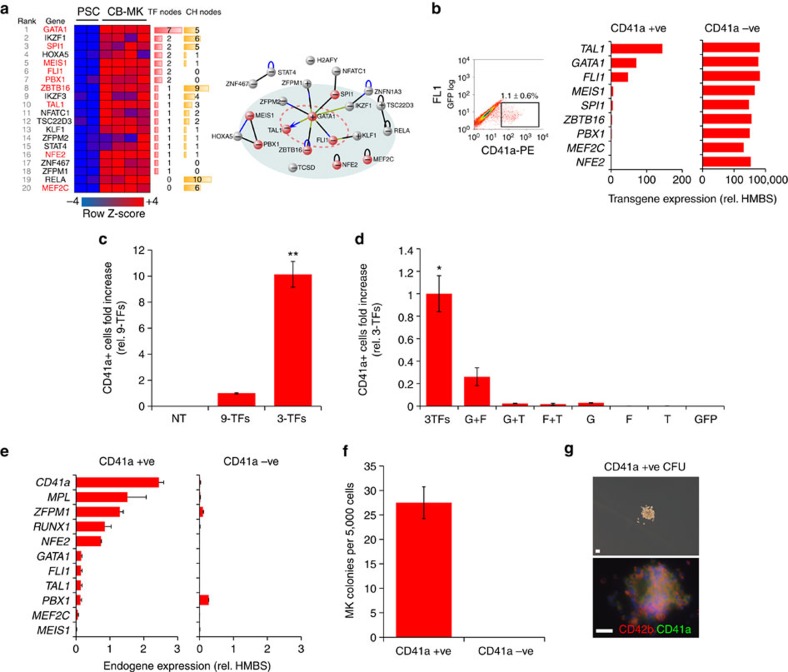
TF candidate screening for MK forward programming. (**a**) TF expression in hPSCs versus cbMKs plotted as a row normalized heatmap with indications of recorded TF internal and chromatin modifiers node numbers and protein interactions among the top 20 TF candidates reported by VisANT. The nine experimentally tested TFs are highlighted in red. (**b**) The H9 hESC line was concurrently transduced with the 9-TFs and maintained in pluripotency medium (FGF2+Activin-A) for 2 days followed by MK medium (TPO+SCF) for a further 5 days. CD41a+ cells generated 7 days after lentiviral transduction (dot plot, mean% ±s.e.m., *n*=5; FL1: 530/40 nm channel) were sorted by flow cytometry and transgene expression levels quantified by RT-qPCR (*n*=1). (**c**) The percentage of CD41a+ cells was monitored by flow cytometry 7 days after transduction of the hiPSC lines #1 and #2 with the 9-TFs or 3-TFs combination. Bar graphs represent the fold increase of CD41a+ cell count relative to the 9-TFs combination (mean ±s.e.m., *n*=4; **P<0.01 by two-tail *t*-test). NT: non-transduced cells. (**d**) The hiPSC#1 line was transduced with all permutations of the 3-TFs and percentages of CD41a+ cells measured by flow cytometry at day 7. Bar graphs represent the fold increase of CD41a+ cell count relative to the 3-TFs combination (mean ±s.d., *n*=2; *P=0.06 and *P<*0.01 versus G+F and other combination respectively by two-tail *t*-test). G: GATA1, F: FLI1, T: TAL1, GFP: control vector. (**e**) The endogenous expression of key MK genes was monitored by RT-qPCR from CD41a flow sorted cells 7 days after transduction of the hiPSC lines #1 and #2 (mean ±s.d., *n*=2). (**f**) The hiPSC lines #1 and #2 were transduced with the 3-TFs and sorted by flow cytometry for expression of CD41a at day 7. The clonogenic potential of sorted cells was tested in methylcellulose semi-solid medium supplemented with TPO and SCF. The number of colonies per 5,000 sown cells was determined after 10 days from duplicate wells (mean±s.e.m., *n*=4). (**g**) A representative MK colony obtained from a CD41a+ cell co-expressing CD41a and CD42b as detected by immunofluorescence is shown (scale bar, 50 μm).

Based on the knowledge that cooperative binding of TFs to target sites can activate gene expression in repressed chromatin^22^, we hypothesized that MK-specific TFs forming complexes able to interact with chromatin remodelers would be able to rewire the hPSC gene regulatory network to induce MK-FOP. We compared TF expression in the H9 hESC line and cord blood-derived megakaryocytes (cbMKs)^23^ and narrowed down to a list of 46 TF candidates subsequently ranked based on differential gene expression level and reported protein interactions between candidates and epigenome modifiers using VisANT^24^ (see Supplementary Fig.1 and Methods for details). Nine TFs from the top 20 candidates were individually cloned into a lentiviral vector backbone under the control of an EF1α promoter to assess their MK-FOP potential (Fig. 1a).

H9 cells were transduced concurrently with equal amounts of each lentiviral vector and subsequently maintained in hPSC medium (FGF2+Activin-A) for 2 days followed by MK medium (thrombopoietin (TPO)+SCF (stem cell factor)) for a further 5 days. The transduction efficiency measured with a green fluorescent protein (GFP) reporter vector was 60.3±5.6% in these conditions (*n*=4). Transduction with nine TFs generated a well-defined population of cells expressing CD41a (integrin alpha-IIb: ITGA2B) on day 7 (1.1±0.6%; Fig. 1b). In an attempt to identify the critical TFs responsible for the generation of this population expressing the marker typically associated with MK lineage commitment, CD41a+ cells were sorted and transgene expression measured by RT-qPCR (quantitative PCR with reverse transcription). Intriguingly, we detected a striking bias for *GATA1*, *FLI1* and *TAL1* (hereafter ‘3-TFs’) transgene expression in the CD41a+ population compared with its negative counterpart which suggested their instrumental role in the acquisition of the CD41a phenotype (Fig. 1b). Accordingly, the 3-TF combination tested in two hiPSC lines generated more CD41a+ cells compared with all nine TFs together demonstrating its superior efficiency (2.5±0.04% versus 0.2±0.04% respectively; Fig. 1c). We assessed all permutations of the 3-TFs and showed that the maximum CD41a+ cell yield was achieved upon concurrent transduction of the 3-TFs which also correlated with a higher clonogenic potential (Fig. 1d; Supplementary Fig. 2a). Importantly, no CD41a+ cells were detected after transduction with a GFP control vector demonstrating that culture conditions were not inductive *per se* (Fig. 1d). Finally, we combined the 3-TFs using a single lentiviral vector carrying a polycistronic expression cassette encoding for the 3-TFs and a GFP reporter. Virtually all GFP-positive cells differentiated into CD41a+ cells by day 7, confirming the efficacy of the 3-TFs in inducing MK-FOP (Supplementary Fig. 2b).

Additional analyses confirmed that the emerging CD41a+ population at day 7 truly represented early MK lineage commitment. We first showed that the expression of key MK genes including *MPL* (coding for the TPO receptor), the TFs *ZFPM1*, *RUNX1*, *NFE2* as well as the endogenous expression of *GATA1*, *FLI1* and *TAL1* were specifically induced in the CD41a+ cell population (Fig. 1e). Moreover, the MK clonogenic potential was exclusively retained in the CD41a+ cell population which further developed to mature MK colonies expressing CD42b (glycoprotein-Ib: GPIBA) functionally demonstrating MK commitment (Fig. 1f,g). Altogether, we thereby identified GATA1, FLI1 and TAL1 as a minimal and sufficient combination of TFs to induce the formation of MK precursors from hPSCs.

### Chemically defined MK-FOP generates high purity MK cultures

**Figure 2:**
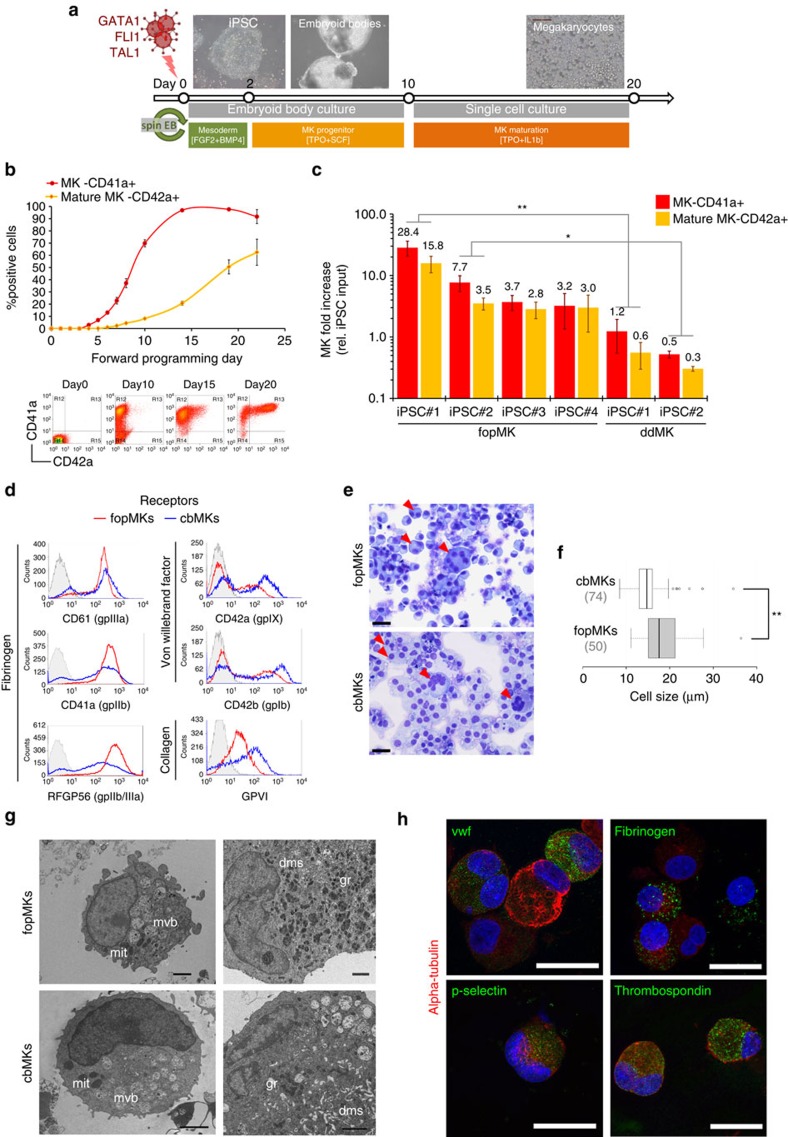
Generation of mature MKs by forward programming using chemically defined conditions. (**a**) Schematic representation of the optimized MK-FOP protocol. Viral transduction at day 0 concurrent with embryoid body generation and mesoderm induction for 2 days was followed by a period of culture in an MK induction medium (TPO+SCF) for 8 days. Embryoid bodies showing cystic structures and actively growing cell aggregates were dissociated to single cells at day 10 and further differentiated to mature MKs (TPO+IL1β) until day 20 post transduction. (**b**) Time course of fopMK differentiation showing MK lineage commitment (%CD41a+ cells) and MK maturation (%CD42a+ cells) from whole culture (mean±s.e.m. from hiPSC lines #1 and #2; *n*=2 (day0–10); *n*=6 (day14–22)). Representative flow cytometry dot plots for CD41a and CD42a expression are shown below. (**c**) The MK fold increase at day 20 relative to the day 0 hiPSC input is shown on a logarithmic scale for the hiPSC lines #1–4 differentiated by forward programming (fopMK: mean±s.e.m.; *n*=14, 7, 3, 5 respectively) or directed differentiation (hiPSCs#1 and #2; ddMK: mean±s.e.m.; *n*=3,2 respectively). **P<0.01 and *P<0.05 by two-tail *t*-test. (**d**) Representative histograms of the expression of major platelet receptors detected by flow cytometry are shown for day 20 fopMKs (red line) and day 10 cbMKs (blue line) against isotype control (grey shade). (**e**) The morphology of day 20 fopMKs and day 10 cbMKs was analysed by modified Romanowsky staining on fixed cells. Arrowheads point to multinucleated cells. Scale bars, 25 μm. (**f**) Cell size distribution from fopMK and cbMK cultures is shown as box plots: centre lines show the medians; box limits indicate the 25th and 75th percentiles; whiskers extend 1.5 times the interquartile range from the 25th and 75th percentiles, outliers are represented by dots (*n*=50, 74 respectively; **P<0.01 by two-tail *t*-test). (**g**) Cell ultrastructure of fopMKs and cbMKs was visualized by transmission electron microscopy. dms, demarcation membrane system; gr, granules; mvb, multi-vesicular body; mit, mitochondria. Scale bars, 2 μm. (**h**) Representative confocal pictures of fopMKs immunostained for major alpha-granule proteins (thrombospondin, fibrinogen, P-selectin and Von Willebrand Factor; iPSC#3 fopMKs, day 40). Scale bars, 25 μm.

To facilitate transfer to the clinic, we developed a chemically defined xeno-free 3-TF MK-FOP protocol. Induction of mesoderm commitment from hiPSCs by a 2-day exposure to FGF2, BMP4 and LY-294002 (ref. 25) significantly increased the percentage of CD41a+ cells at day 7 compared with pluripotency maintenance conditions (5.4±1.6-fold increase; Supplementary Fig. 3a). Next, we assessed the benefit of using forced aggregation embryoid body formation instead of the base two-dimensional (2D) culture. When coupled with mesoderm-inducing conditions, embryoid body culture further improved the yield of CD41a+ cells at day 7 (5.2±0.9-fold increase; Supplementary Fig. 3b). The embryoid body culture had the additional advantage of providing a standardized quantity of input cells thereby conferring reproducibility. The final protocol combined lentiviral transduction with embryoid body formation over the first 24 h with mesoderm induction for the first 2 days. This protocol allowed consistent transduction efficiencies amongst hiPSC lines (68.2±4.1% using a single GFP control vector, *n*=11 using three hiPSC lines), resulting in 22% co-transduction efficiency as estimated using reporter vectors (Supplementary Fig. 3c). As MK maturation was not optimally sustained in the low-adherence embryoid body culture setting, we included a single-cell dissociation step which gave an optimal MK yield when performed at day 10 post transduction (Supplementary Fig. 3d) followed by a further 10 day culture in medium containing TPO and IL1β routinely used for cbMK differentiation^26^. The optimized MK-FOP protocol using xeno-free GMP-grade basal medium and recombinant cytokines is depicted in Fig. 2a.

We observed a gradual increase in CD41a+ cells from day 4 post transduction followed by the acquisition of the mature MK marker CD42a (glycoprotein IX:GP9, component of the Von Willebrand platelet receptor complex) from day 6 onwards mimicking cbMK differentiation (Fig. 2b; Supplementary Fig. 3e). Critically, using two different hiPSC lines, MK-FOP consistently achieved MK lineage purity (>95% CD41a+ cells) by day 15 post transduction with >50% CD42a+ mature MKs by day 20 (Fig. 2b). A robust cell expansion during the single-cell culture step led to the generation of large quantities of MKs with up to 28.4±7.8-fold increase relative to the hiPSC input at day 20 (iPSC#1, *n*=14; Fig. 2c). Interestingly, MK-FOP achieved significantly higher cell yields compared with a standard MK-directed differentiation approach^27^, producing in our hands on average 26.3 and 11.7 times more mature MKs from the hiPSC lines #1 and #2, respectively (Fig. 2c). In addition, the MK purity obtained by MK-FOP was significantly higher than MK-directed differentiation (97.7±0.8% versus 21.3±2.9%, respectively; Supplementary Fig. 3f).

To further assess the MK identity of FOP cells, day 20 fopMKs were compared with day 10 cbMKs as a benchmark. At this stage, both cultures show similar MK maturity (50–60% CD42a+ cells; Fig. 2b; Supplementary Fig. 3e). The key platelet surface receptors for fibrinogen (αIIb-β3), Von Willebrand factor (gpIb-V-IX) and collagen (gpVI) were readily detected in both cultures (Fig. 2d). Morphologically, fopMK cultures displayed a typical mix of megakaryoblasts with large peripheral nuclei and mature MKs with increased cytoplasmic volume and frequent polyploid cells similarly to cbMK cultures (Fig. 2e,f). In accordance with published results for *in vitro*-derived neonate and hPSC MKs^17^,^28^, the cell ploidy remained low on average and was comparable for both fopMKs and cbMKs (<2% 8*N* cells; Supplementary Fig. 3g). Analysis of the fopMK ultrastructure by electron microscopy showed lobulated nuclei, developing demarcation membrane system (cytoplasmic cell membrane supply for platelet release), cytoplasmic multi-vesicular bodies (precursors to platelet granules) and mature granules (Fig. 2g). We confirmed by confocal microscopy that major alpha-granule proteins (P-selectin, thrombospondin, fibrinogen and vWF) were indeed correctly expressed and patterned in fopMKs (Fig. 2h). Importantly, the total expression level of the 3-TFs in fopMKs—as sum of transgenic and endogenous expression—was not significantly different from cbMKs (Supplementary Fig. 3h). Collectively, these data demonstrate the efficient production and maturation of MKs derived by forward programming in xeno-free chemically defined conditions.

### MK-FOP produces expandable and cryobankable mature MKs

**Figure 3:**
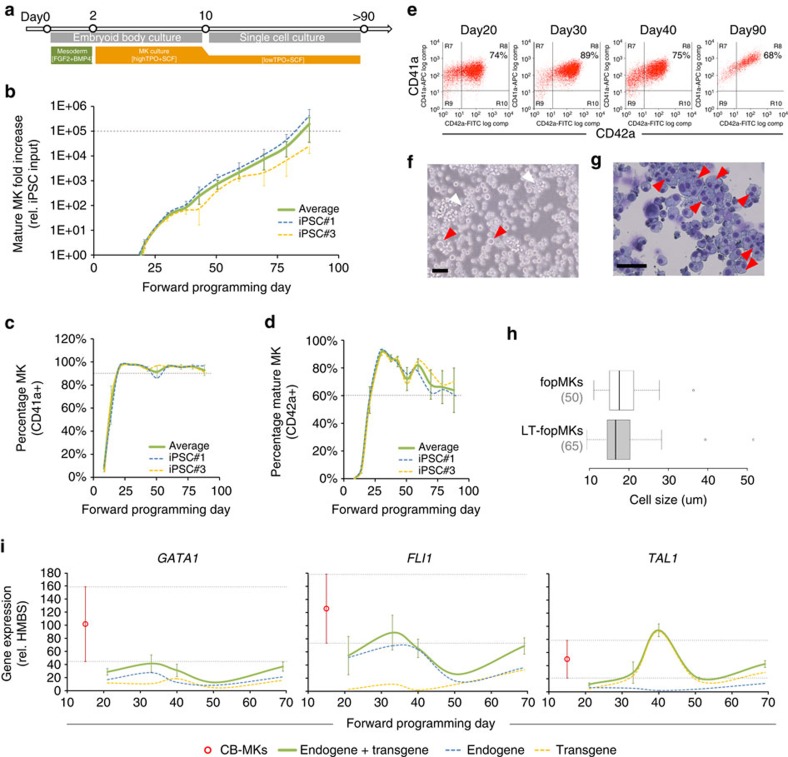
Long-term expansion of fopMKs. (**a**) Schematic representation of the culture conditions for long-term MK-FOP. The combination of TPO (20 ng ml^−1^) and SCF (50 ng ml^−1^) allowed mature MK expansion for 90 days and beyond. (**b**) The cumulative mature MK (CD41a/CD42a double positive cells) fold increase relative to day 0 hiPSC cell input is shown for the hiPSC lines #1 (*n*=4) and #3 (*n*=5) over 90 days in culture (mean±s.e.m.). (**c**,**d**) The corresponding percentages of CD41a+ and CD42a+ cells monitored by flow cytometry from whole cultures are shown over the 90 day period (mean±s.e.m.). (**e**) Representative flow cytometry dot plots for CD41a and CD42a expression from the hiPSC#3 line. (**f**,**g**) Representative phase contrast picture of a day 70 hiPSC#3 fopMK long-term culture and associated Romanowsky staining of fixed cells (scale bars 50 μm). White arrowheads: clumps of actively growing small cells; red arrowheads: single big cells in suspension culture and polyploid cells identified by Romanowsky staining. (**h**) Cell size distribution from fopMK and LT-fopMK cultures is shown as box plots (*n*=50, 65 respectively). (**i**) Endogenous and transgenic expression levels of the 3-TFs were independently monitored by RT-qPCR throughout MK-FOP long-term cultures (mean±s.e.m. from hiPSC lines #1 and #3; *n*=3). The average range of expression levels in cbMKs (mean±s.e.m.; *n*=5) is shown as a benchmark (in red).

We found that fopMKs could be maintained in culture and kept expanding for an extended period of up to 60 days in the TPO+IL1β condition described above (Supplementary Fig. 4a–c). The expression of KIT (the receptor for SCF, an haematopoietic progenitor pleiotropic cytokine) on a fraction of the cells suggested the persistence of a progenitor population in MK-FOP cultures (Supplementary Fig. 4d). This led us to test the continuous supplementation with SCF (50 ng ml^−1^)—instead of IL1β originally used through the second step of culture—in an attempt to improve further long-term maintenance. In addition, we reasoned that the high TPO level (100 ng ml^−1^) may not be required in the MK-FOP context where differentiation was sustained internally by expression of the 3-TFs, and indeed high TPO may be responsible for precocious exhaustion of MK-FOP cultures by over stimulation of differentiation. Consequently, we tested a lower TPO concentration (20 ng ml^−1^) in combination with SCF through the second step of MK-FOP (Fig. 3a). In these conditions, we were able to maintain fopMKs in culture with steady proliferation for at least 90 days, achieving close to an average 200,000 MK yield per input hiPSC (1.94±1.59 × 10^5^, *n*=7 for hiPSC#1 and #3 cumulatively; Fig. 3b). This was in striking contrast with the maximum 1,300 MK fold increase, earlier loss of CD42a expression and cell viability in long-term culture using the original high TPO and IL1β condition (Supplementary Fig. 4a–c). The MKs harvested from optimized long-term cultures (>30 days, defined thereafter as LT-fopMKs) maintained a purity of over 90% CD41a+ cells with levels of CD42a expression >60% (Fig. 3c–e) and an increase in late maturation markers like GPVI (Supplementary Fig. 4e). The LT-fopMK cultures contained a mixed population of small megakaryoblasts growing in loose clusters representing the actively proliferating cell fraction together with larger polyploid MKs (Fig. 3f–h). Critically, LT-fopMKs were successfully frozen over a day 21–70 timeframe and subsequently recovered for further culture and expansion allowing cryobanking of fopMK batches (Supplementary Fig. 4f).

We found that LT-fopMK cultures were not immortalized but finite with the longest iPSC#1 and iPSC#3 cultures kept for 120 and 132 days, culminating respectively in 17 million and 800,000 MK fold increases (*n*=4/5 biological replicate respectively; Supplementary Fig. 4g). Beyond day 90, most LT-fopMK cultures showed a drastic loss of cell viability (Supplementary Fig. 4h) associated with a steady decrease of clonogenic potential and of CD34+ haematopoietic progenitor content (Supplementary Fig. 4i,j). At the population level, the average expression of the 3-TFs (sum of endogenous and transgenic expression) in LT-fopMK cultures was maintained in a close range to day 10 cbMKs (Fig. 3i). Together, we showed the 3-TF-driven MK-FOP generated a highly proliferative albeit exhaustible progenitor pool sustaining the expansion of mature MKs *in vitro* for over 3 months.

### Genome-wide analysis confirms long-term MK identity

We analysed the whole-genome microarray expression data of MKs derived *in vitro* by different protocols and from different stem cell sources: by FOP or directed differentiation from hiPSCs (fopMKs, LT-fopMKs and ddMKs) and from cbMKs, all samples with purity greater than 95% CD41a+ and 80% CD42a+ cells.

**Figure 4:**
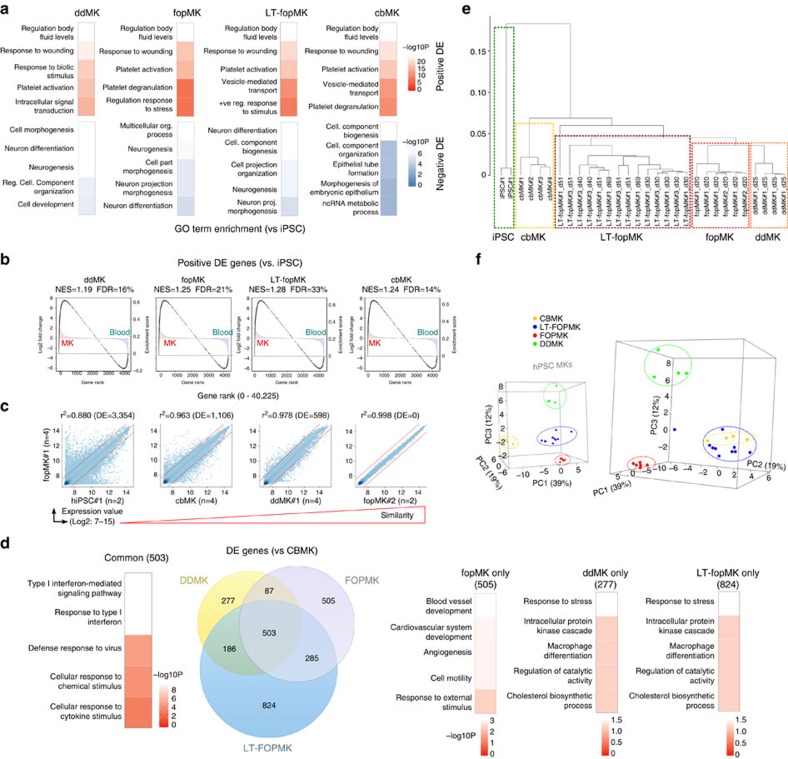
Transcriptional landscape of forward programmed MKs. The undifferentiated hiPSC#1 line (*n*=2), day 20 fopMKs (hiPSC#1–2, *n*=4,2), day 25 ddMKs (hiPSC#1, *n*=4), day 10 cbMKs (*n*=4; all three groups CD42b+ sorted >95%) and day 30–69 LT-fopMKs (hiPSC#1 and #3, *n*=8 and 6; >80% CD42b+), were analysed for gene expression using Illumina Human HT-12 v4 BeadArrays. (**a**) Top five enriched gene ontology biological processes for differentially expressed (DE) genes in all MK samples compared to hiPSCs. −Log_10_ *P* values are shown as colour scale. (**b**) Gene set enrichment analyses for DE genes from the different MK samples (versus hiPSCs; grey circles) against a ‘MK versus other blood types’ gene expression data set (from Haematlas^23^). NES, normalized enrichment score; FDR, false discovery rate. (**c**) Gene expression correlation scatter plots on the whole-gene set using pairwise comparisons of different MK groups. R^2^ Pearson correlation value and differentially expressed gene numbers are indicated. The differential expression threshold (two-fold-change; FDR 5%) is shown as dotted red lines. (**d**) Venn diagrams recording DE genes (|Log2 fold-change| >1; FDR5%) in hiPSC-derived MKs compared with cbMKs. The number of DE genes is indicated for each intersection and the top five enriched gene ontology term biological processes from the Venn Diagram intersections are shown. (**e**) Hierarchical clustering using the average agglomerative method on whole-gene data set. (**f**) Three-dimensional plots of the principal component analysis of MK populations. The first 3 PC are shown with respective percentages of variance indicated in brackets. The two principal component analysis boxes are snapshots of a rotation along the PC3 axis with MK sample groups highlighted.

First, enrichment analysis using hyperG test (false discovery rate (FDR) ≤5%) of gene ontology terms from upregulated genes versus undifferentiated hiPSC confirmed that MK-FOP efficiently induced the MK phenotype with top biological processes in fopMKs and LT-fopMKs related to haemostasis/platelet gene ontology terms as was the case for ddMKs and cbMKs (Fig. 4a). This was mirrored by a depletion in differentiation/morphogenesis gene ontology term-associated genes indicating appropriate downregulation of pluripotency features (Fig. 4a). Moreover, the gene set enrichment analysis^29^ against a panel of blood cells (cbMKs versus other blood cells from Haematlas^23^; *n*=4 and 46, respectively) showed specific enrichment of MK-specific genes in the list of genes upregulated in fopMKs further demonstrating the acquisition of a genuine MK phenotype (normalized enrichment score (NES)=1.25, FDR=21%; Fig. 4b). The highly similar expression profiles obtained from two distinct hiPSC lines demonstrates the inter-line qualitative reproducibility of MK-FOP (*r*^*2*^=0.998 for iPSC#1 and #2; Fig. 4c).

Differential expression analysis comparing hiPSC-derived MKs to cbMKs revealed a number of differential expression genes that were (in accordance with the MK phenotype acquired by fopMKs) not related to haemostasis/platelet-related biological processes (503 common, 2,667 total; ≥2-fold-change and FDR≤5% cutoff; Fig. 4d). Hierarchical clustering distinguished the four MK groups while showing a separate cluster encompassing the hiPSC-derived MKs (i.e., fopMKs, LT-fopMKs and ddMKs) distinct from cbMKs indicative of an intrinsic difference resulting from their hPSC provenance, as previously described^30^ (Fig. 4e). Interestingly, while confirming a shared hiPSC-MK identity distinct from cbMKs, the principal component analysis indicated that LT-fopMKs clustered closer to cbMKs than fopMKs and ddMKs (Fig. 4f)^30^. Altogether, the whole-genome expression analysis validated the acquisition by MK-FOP of a genuine MK phenotype that was effectively maintained throughout long-term culture.

### *In vitro* production of functional platelets by fopMKs

**Figure 5:**
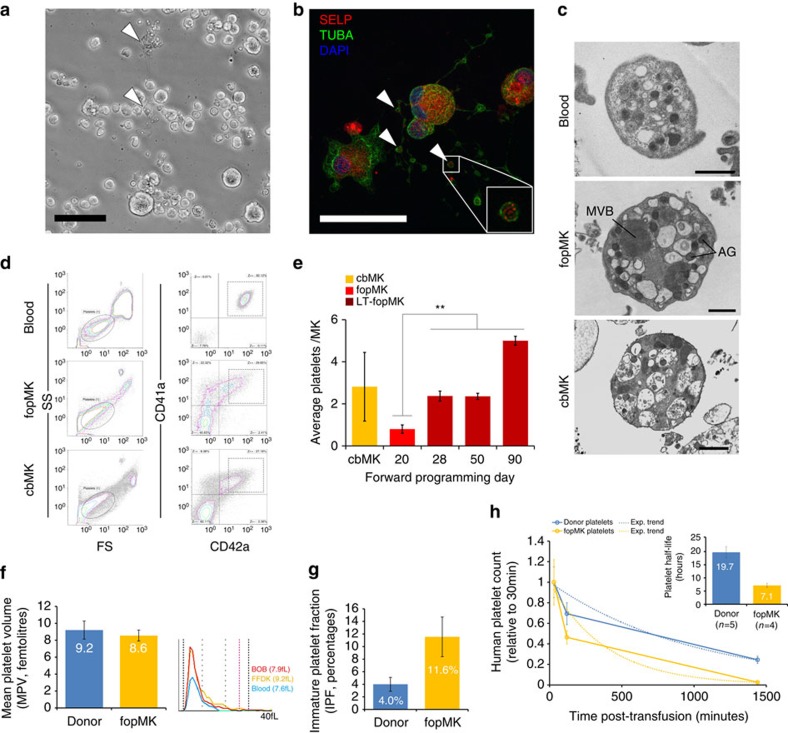
Platelet release *in vitro* from fopMKs throughout long-term culture. (**a**) Phase contrast picture of spontaneous proplatelet-forming fopMKs (arrowheads) in suspension culture (hiPSC#3, day26; scale bar, 50 μm). (**b**) Proplatelet-forming fopMK on fibrinogen-coated slide immunostained for alpha-tubulin (TUBA) and P-selectin (SELP). Arrowheads indicate nascent platelet tips containing SELP-positive granules (hiPSC#4, day21; scale bar, 50 μm). (**c**) Transmission electron microscopy pictures of blood and *in vitro*-produced platelets showing typical ultrastructure. AG, alpha-granules; MVB, multi-vesicular bodies (scale bars, 1 μm). (**d**) Representative flow cytometry dot plots of platelet analysis. Platelets are defined within the human platelet size gate as CD41a/CD42a double positive events. (**e**) cbMK and fopMK from different culture time points were sown on C3H10T1/2 feeder cells for 48 h and the number of platelets released in the supernatant quantified by flow cytometry. Data represent the mean±s.e.m. of platelet number per MK sown (*n*=7, 4, 10, 6, 2 for cbMK and fopMK (hiPSC#1–4 pool) at day 20–90 respectively; **P<0.01 versus fopMK day 20 by two-tail *t*-test). (**f**,**g**) Mean platelet volume (MPV) ±s.d. and immature platelet fraction (IPF) ±s.d. of washed donor and fopMK platelets as measured on a Sysmex whole-blood analyzer (*n*=4/2 respectively; hiPSC#1 and #5). (**h**) Human platelet survival in NSG mice circulation over 24 h measured by flow cytometry following the systemic injection of 20 million washed platelets. The exponential trend of human platelet absolute count decrease over time was used for half-life calculation (mean±s.e.m.; *n*=5/4 for donor and fopMK (hiPSC#1 and #5) platelets respectively).

Mature MKs produce platelets by a process of proplatelet formation whereby MKs extend cytoplasmic protrusions into the bone marrow blood stream^31^. These protrusions contain multiple branching points and bulbous ends with active accumulation of granules into the end processes that represent the nascent platelets, which then mature further in the circulation. In culture, fopMKs formed proplatelets containing P-selectin-positive α-granules (Fig. 5a,b; Supplementary Movies 1 and 2). For further analyses, *in vitro* platelet production was maximized using a static co-culture system with the murine C3H10T1/2 feeder cell line as previously described^27^. Electron microscopy showed that platelets produced *in vitro* from fopMKs and cbMKs, while heterogeneous in quality reflecting the current limitations of 2D static cultures, showed the typical platelet ultrastructure notably including high alpha-granule content (Fig. 5c). We further used flow cytometry for the quantitative measurement of *in vitro* platelet production from fopMKs, strictly defining platelets as CD41a+/CD42a+ particles of human platelet size (Fig. 5d). A significant increase in platelet production from LT-fopMKs versus day 20 fopMKs was observed matching platelet release from cbMKs (5±0.2 versus 0.8±0.2 platelets per MK at day 90 and day 20, respectively; Fig. 5e). The platelet production rate was similar amongst fopMKs derived from different hiPSC lines (hiPSC#1–4; Supplementary Fig. 5a). *In vitro* platelets showed surface expression of the main thrombocyte receptors including the GPIIb/IIIa complex (fibrinogen receptor), GPIb and GPIX (Von Willebrand factor receptor), GPIIa and GPVI (collagen receptors), some of them with a decreased intensity compared with donor platelets which has been previously described and likely originated from the static 37 °C *in vitro* culture conditions used for production (Supplementary Fig. 5b)^20^,^32^. The fopMK platelets showed a normal mean volume on a clinical Sysmex blood analyzer (8.6±0.7 fl, *n*=2, iPSC#1 and #5; Fig. 5f), and interestingly an increased immature platelet fraction compared with normal circulating blood (11.6±3% versus 4±1%, respectively; Fig. 5g). Eventually, we assessed fopMK platelet survival *in vivo* in immunodeficient NOD scid gamma (NSG) mice with further splenic macrophage depletion to allow human platelet maintenance in the circulation^33^. The fopMK platelets were readily detected in the circulation for several hours while showing a shorter half-life than primary donor platelets (7.1±0.8 h versus 19.7±2.2 h, respectively; Fig. 5h), a result which was very similar to previously published data (7.5 and 18.3 h, respectively)^19^ and probably biased by the limitations of the current static *in vitro* production systems for the generation of homogenous populations of genuine resting platelets endowed with longer circulation half-life. To further distinguish functional platelets from the heterogeneous *in vitro*-produced pool, we used Calcein-AM as a marker of platelet viability and membrane integrity^34^. The proportion of Calcein-AM-positive platelets within fopMK- and cbMK-derived platelet harvest was 32.4±0.8% (*n*=3; iPSC#1, #5) and 40.1±3.6%, respectively (*n*=3) and defined a phenotypically more homogenous CD41a+/CD42a+ platelet population similar to control blood (Supplementary fig. 5c). Hereafter, *in vitro* generated platelets were identified using Calcein-AM staining to compare their function with donor-derived platelets.

**Figure 6:**
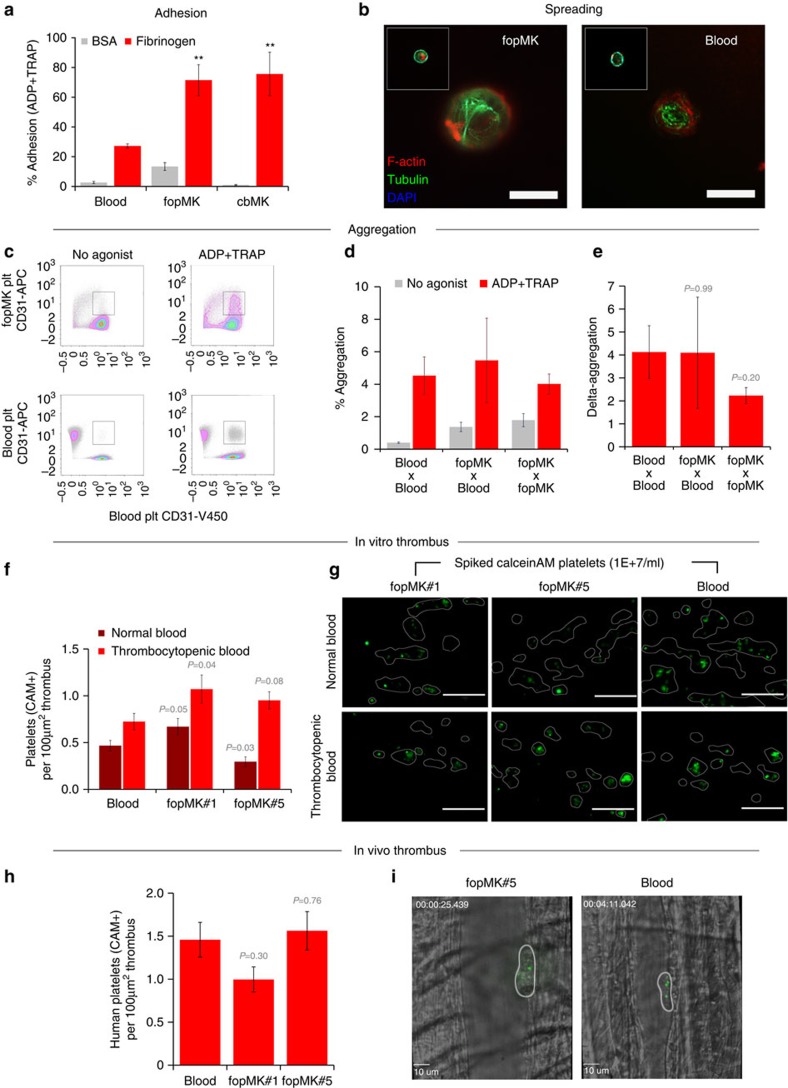
Functional assessment of fopMK *in vitro* platelets. (**a**) Adhesion to fibrinogen of fopMK and cbMK *in vitro* platelets upon combined TRAP+ADP stimulation compared to blood platelets using a flow cytometry bead based assay. Percentages of adhesion on BSA or fibrinogen-coated beads are shown (mean±s.e.m., *n*=4, 4, 2 for blood, fopMK and cbMK respectively; **P<0.01 versus blood by two-tail *t*-test). (**b**) Representative pictures from *in vitro s*preading assay. Washed platelets were sown on fibrinogen-coated slides, incubated for 45 min at 37 °C and immunostained for alpha-tubulin (TUBA) and F-actin (scale bars, 10 μm). (**c**) Aggregation of fopMK platelets upon agonists stimulation was tested both with and compared with blood platelets using a flow cytometry-based assay: representative dot plots are shown. (**d**) Percentages of aggregation from Calcein-AM+ live platelets upon stimulation are shown for blood and fopMK platelets reactions (2 × 10^7^ platelets per ml; mean±s.e.m., *n*=7 for each reaction group; no statistical difference versus blood at *P<*0.01 by two-tail *t*-test). (**e**) Associated delta-aggregation defined as ((% ADP+TRAP aggregation)—(% no agonist aggregation)) for the different reaction groups (mean ±s.e.m., *n*=7; *P* values by two-tail *t*-test versus blood indicated). (**f**) Thrombus formation *in vitro* under arterial shear stress. The participation of Calcein-AM live platelets spiked into human blood (at 1 × 10^7^ per ml) is shown per 100 μm^2^ thrombus area. Normal or thrombocytopenic blood (>150 × 10^9^ and <50 × 10^9^ l^−1^ respectively) was used as recipient. Spiked platelets were sourced from day-8 concentrate unit (blood) or from fopMK platelets (iPSC#1 and #5; *n*>30 analysed thrombi per group; *P* values by two-tail *t*-test versus blood indicated). (**g**) Representative pictures from *in vitro* thrombus formation assays. Thrombi identified using bright field images are delineated and Calcein-AM platelets fluorescing in green; *in vitro* platelets Calcein-AM labelling is intrinsically dimmer than donor-derived platelets (Supplementary Fig. 5b). Scale bar, 50 μm. (**h**) Thrombus formation *in vivo* by laser injury of an arteriole in the cremaster muscle of NSG mice and intravital confocal microscopy. The incorporation of human Calcein-AM-labelled platelets (50 million transfused per mouse) to mouse thrombi is shown per 100 μm^2^ thrombus area at T_max (thrombus maximum size). Mean values±s.e.m. and *P* values by two-tail *t*-test versus donor platelets are shown (*n*=16/4/8 thrombi analysed for blood, fopMK#1 and #5 platelets respectively). (**i**) Representative snapshots of Calcein-AM+ human platelets incorporated to mouse thrombus (scale bar 10 μm).

To fulfil their haemostatic role, platelets must be able to sequentially adhere to damaged vessels (using collagen and other extra-cellular matrix receptors), increase their surface by spreading (by active remodelling of their cytoskeleton), build-up the thrombus by aggregation of other platelets (through fibrinogen binding) and eventually amplify the haemostatic response through degranulation (granule content release to the surface)^35^. We first compared adhesion with fibrinogen using a quantitative flow cytometry assay^36^. Similarly to platelets generated *in vitro* from cbMKs, an increased adhesion to fibrinogen was observed for fopMK-derived platelets as compared with blood platelets (Fig. 6a). This distinction may be inherent to the platelet harvest methodology or originate from intrinsic developmental biological differences of hiPSC and neonates platelets compared with adult blood platelets^37^. We further showed that fopMK-derived platelets spread efficiently upon contact with fibrinogen presenting typical tubulin cytoskeletal reorganization (Fig. 6b). We then used a flow cytometry approach to quantitatively measure agonist-induced platelet aggregation^38^ (Fig. 6c). We showed that in response to agonist stimulation (a combination of ADP and TRAP), fopMK-derived platelets efficiently aggregated with fresh blood platelets as well as between themselves with no significant difference in aggregation potential as compared with blood platelets (Fig. 6d,e). Eventually, we performed thrombus formation *in vitro* under physiological shear stress as a high-level assay of integrated platelet functions. To mimic a transfusion setting, we introduced a defined amount of Calcein-AM-labelled *in vitro*-produced platelets or blood platelets from a transfusion unit into normal or thrombocytopenic fresh human blood (platelet count <50 × 10^9^ per litre; spiked platelets at 10 × 10^9^ per litre; Supplementary Movies 3 and 4). When exposed to collagen under arterial shear rates (1,600 s^−1^), fopMK-derived platelets took part in clot formation similarly to platelets from a concentrate unit (Fig. 6f,g). We further observed an increase in the contribution of fopMK platelets to the thrombi in the context of thrombocytopenic blood (Fig. 6fg; Supplementary Fig. 5d). Furthermore, P-selectin surface expression was observed in the platelets participating in the thrombi, thereby proving adequate degranulation of activated fopMK platelets (Supplementary Fig. 5e). The validation of fopMK platelet function was ultimately addressed *in vivo* using intravital microscopy and laser injury-induced thrombus formation in the cremaster vasculature of NSG mice^39^. The transfusion of 5E+7 Calcein-AM-labelled platelets into mouse circulation allowed the visualization of human platelet integration into thrombi with comparable efficiencies between donor and fopMK platelets (1.5±0.2 and 1.4±0.2 platelets per 100 μm^2^ thrombus area respectively, *n*=16/12; Fig. 6h,i). Strikingly, *in vitro*-produced fopMK platelets showed active participation in clot formation with visible sequential rolling, binding, thrombus surface surveying and spreading (Supplementary Movies 5 and 6).

While current systems for *in vitro* platelet production from cultured MKs can be further optimized beyond the 2D static approach used here, platelets produced *in vitro* from fopMKs were nevertheless functionally similar to donor-derived platelets across a range of assays, thereby affirming their suitability and promise for a host of regenerative medicine research and therapeutic applications.

## Discussion

We describe here forward programming, a novel approach for the chemically defined large-scale production of MKs from hiPSCs. We drive MK development by the combined ectopic expression of three TFs: GATA1, FLI1 and TAL1. These three TFs have well-documented roles in haematopoiesis, especially in the maintenance of early haematopoietic progenitors, red blood cell and megakaryocyte differentiation^40^,^41^,^42^,^43^,^44^. Forced expression of GATA1 or TAL1 alone in haematopoietic progenitors has been shown to bias differentiation towards MK and erythroid fates^45^,^46^, while FLI1 cooperates with GATA1 to enable MK maturation^47^. In addition, TAL1 and FLI1 play an earlier role in the specification of the haematopoietic programme during vertebrate embryonic development^48^,^49^. Accordingly, exogenous expression of TAL1 in hESCs has been reported to promote haematopoiesis and megakaryopoiesis^50^,^51^. More recently, the combinatorial expression of TAL1 with GATA2 was found to induce an hemogenic endothelial phenotype biased towards erythro-megakaryocytic differentiation from hPSCs^52^. Nevertheless, the MK-FOP approach described here involving co-expression of the 3-TFs GATA1, FLI1 and TAL1 is unprecedented in regard to its efficiency in rapidly imposing MK progenitor identity to hPSCs in very stringent MK-specific culture conditions. The high hierarchical status of the 3-TFs within the MK gene regulatory network, already supported by previously published gene interaction network and ChIP-sequencing data in primary human MKs^26^,^53^ (Supplementary Fig. 2c), is now functionally confirmed in human cells by the efficiency of the MK-FOP approach.

It remains to be demonstrated how closely the 3-TF programming recapitulates normal haematopoietic development from hPSCs. Current data indicate that mesoderm commitment is strongly beneficial to MK-FOP, consistent with the normal ontogeny of blood cells in the embryo. Intriguingly, we observed an early expression of hemogenic endothelium markers (FLK1, CD34 and VE-Cadherin) from day 2 of programming which was intermingled with expression of the megakaryocyte commitment marker CD41a and quickly followed by a marker of blood commitment with the increasing concurrent CD43 expression from day 3 onwards (Supplementary Fig. 6). The progressive decrease of endothelial populations associated with a steady TPO-dependent growth of CD41a+ cells from day 4, as well as the absence of monocyte/granulocyte colonies in clonogenic assays, indicates an early commitment to MK fate (Supplementary Fig. 6). Taken together, these observations suggest that overexpression of the 3-TFs in hPSCs may at least partially recapitulate key steps of the blood ontogeny with a contracted hemogenic endothelium phase and early enforcement of MK identity. Controllable and traceable 3-TF expression systems combined with molecular analysis at the single-cell level will be necessary to further decipher the precise molecular mechanisms governing MK-FOP.

There is high biotechnological significance in our ability to maintain the forward programmed cells in long-term cultures and expand them over time whilst preserving MK purity, markers of maturity and platelet production. This TPO- and SCF-dependent cellular proliferation persisted for an extended but not indefinite timeframe (90–134 days) and is presumably sustained by non-transformed early MK-biased progenitors (<1% by CFU assays) able to proliferate and differentiate further towards mature MKs. In this respect the LT-fopMK culture appears different in nature to the recently described expandable MK cell lines obtained from hPSCs by sequential ectopic introduction of MYC, BMI1 and BCL-XL into MK-committed cells^20^. The generation of these MK cell lines notably requires the precise control of MYC expression levels and subsequent silencing of those three overexpressed genes to achieve full MK maturation. The complex genetic modifications involved and the limited success rate of immortalized MK line derivation—involving manual screening and clonal selection—along with the initial generation of blood progenitors in undefined conditions are limitations of that protocol for both research and clinical development. MK-FOP did not produce an immortalized MK progenitor and did not require manipulation of gene expression levels to produce fully mature MKs. Interestingly, MK lineage biased self-renewing progenitors have been recently identified in the haematopoietic stem cell phenotypic compartment of the bone marrow^54^. Further investigations are needed to understand the full nature and the 3-TF-driven molecular mechanisms underpinning the generation of this long-term expanding MK progenitor.

Recently, another approach has been published that allows the expansion of MK progenitors derived from PSCs^55^. In contrast with MK-FOP, that system is based on the artificially controlled downregulation of GATA1 in haematopoietic progenitors allowing MK progenitor expansion and its restoration to enable subsequent MK maturation. The approach, which was developed with mouse ESCs, has yet to be demonstrated to work in human cells. In addition, the authors did not show efficient platelet production from these MKs *in vitro*, instead choosing an adoptive transfer of MKs into recipient mice. The injection of nucleated cells derived from human pluripotent stem cells would raise crucial issues of potential tumorigenicity that could constrain future clinical use.

The quantitative functional platelet data collected here proved that platelets derived from fopMKs *in vitro* are endowed with major platelet functions allowing efficient thrombus formation as shown in previous studies^19^,^20^. Although abnormalities in key TFs for the MK lineage can lead to abnormal platelet function^43^, the preservation of fopMK platelet function is consistent with the fact that the overall expression levels of the 3-TFs is controlled throughout the programming progression and similar to cbMKs. The remaining bottleneck for application to transfusion medicine is the optimization of platelet generation *in vitro*: this presently remains on average 1,000-fold lower than the *in vivo* platelet yield per MK and is accompanied by issues regarding the purity of the final product where functional platelets represent only a fraction of the platelet size particles in the whole harvest^19^,^20^,^56^. In this study, which focuses on the production of the MKs themselves, we have used a previously published platelet production system based on co-culture with a mouse stromal cell line^27^,^57^. Our findings clearly confer an impetus to achieve further progress using newly developed three-dimensional laminar flow systems and bone marrow mimicking scaffolds^58^,^59^,^60^ by providing ample quantities of functional MKs from hPSCs using a simplified chemically defined protocol amenable to the generation of a clinical product.

In conclusion, our study demonstrates the feasibility of a forward programming approach to generate mature functional MKs from human PSCs that significantly transcends available directed differentiation protocols through a unique combination of key achievements. First, the methodology results in a very high cell yield and MK purity using fully chemically defined xeno-free culture conditions. To put this in the clinical context, the long-term culture expansion allows a cumulative production of 2 × 10^11^ MKs releasing 1 × 10^12^ platelets—the equivalent of 3 transfusion units—starting from only one million hiPSCs. Moreover, the minimal cell handling and cytokine requirements, the cryopreservation of fopMKs enabling cell banking and future stock management and the successful differentiation of an array of hPSC lines with qualitative reproducibility are additional critical strengths of the MK-FOP approach for future manufacturing of platelets for human therapeutic applications. The effectiveness and versatility of MK-FOP opens new avenues for future basic research and functional studies on novel MK and platelet genes as well as disease modelling using hiPSCs^53^,^61^,^62^.

## Methods

### Human pluripotent stem cell culture

The H9 hESC line (WiCell; passages 75–95) and hiPSC lines (iPSC#1–5; A1ATD1, BBHX8, A1ATD1-c, S4-SF5 and FFDK1, respectively, p30–50) were cultivated as clumps in a chemically defined medium (CDM) containing recombinant human FGF2 and Activin-A (15 ng ml^−1^ each, internal) on feeder-free gelatin or vitronectin coated wells as previously described^63^. All hiPSC lines were obtained from the Cambridge Biomedical Research Centre iPSC Core Facility and have been derived from adult dermal fibroblasts using integrative murine retroviral vectors (iPSC#1–2), cytoplasmic Sendai viral vectors (iPSC#3–4) or episomal vectors (iPSC#5) expressing the human OCT4, SOX2, KLF4 and MYC reprogramming factors.

### Selection of transcription factor candidates

We performed a differential gene expression analysis focused on DNA binding protein coding genes (PANTHER Classification System) from whole-genome expression data generated using the H9 hESC line (internal data, Illumina HumanWG-6 v3) and human cord blood-derived MKs^23^. The list of 116 MK-specific genes generated was further refined by removal of 21 histone-coding genes and addition of 6 candidates based on previous knowledge of their role in megakaryopoiesis (Supplementary Fig. 1a). Using the VisANT web-based software^24^, the resulting 101 candidate genes were subsequently ranked based on number of (1) internal protein interactions, (2) interaction with epigenetic modifiers (HAC, HDAC, DNMT, list in Supplementary Fig. 1c) and (3) differential expression levels (Supplementary Fig. 1b). Genes with low differential expression (Log_2_(MK-ESC)<1 or <2 with no reported interactions) were excluded from the candidate list.

### Recombinant lentiviral vectors

#### Transcription factor cloning

The human coding sequences of the nine candidate genes (variants 1 from NCBI Reference Sequence Database) including the 5′ Kozak consensus sequence were generated by PCR using cbMK cDNA, individually cloned into the pWPT lentiviral vector backbone (Dr Trono, Addgene #12255) downstream of the human EF1-alpha ubiquitous promoter and checked for sequence integrity.

#### Viral particle production

Replication deficient lentiviral vector particles (LVPs) were produced by transient co-transfection of HEK 293T/17 cells (ATCC CRL-11268) with pWPT constructs along with the psPAX2 and pMD2.G helper plasmids (Addgene #12260, #12259) using TransIT-LT1 transfection reagent (MirusBIO). Crude supernatants containing LVPs were concentrated by PEG-based precipitation (LentiX-concentrator, Clontech) and functional titres determined by qPCR measurement of provirus copy number in genomic DNA of transduced HCT116 cells (ATCC CCL-247).

#### Human pluripotent stem cell transduction

hPSC lines were routinely transduced by 18–24 h single exposure to LVPs using multiplicity of infection of 20 in presence of 10 μg ml^−1^ Protamine Sulfate (Sigma) in routine culture medium.

### Megakaryocyte forward programming

#### Optimized embryoid body based protocol

On transduction day (day 0), sub-confluent (50–80%) hPSC cultures were dissociated to single cells using TrypLE (Life Technologies) Fig. 2a. Embryoid body formation was initiated with 6–12E+5 viable cells per well of an Aggrewell400 plates (Stem Cell Technologies) leading to 500–1,000 cells per embryoid body following spin aggregation. Lentiviral transduction was performed concomitantly to the aggregation step in CDM supplemented with Y-27632 (10 μM, Sigma), BMP4 (10 ng ml^−1^, R&D) and protamine sulfate. After 24 h, transduced embryoid bodies were collected and sown in ultralow adherent cell culture plates (Corning) at 1,200 embryoid bodies per 10 cm^2^ dish in CDM with BMP4 and FGF2 (5 ng ml^−1^). Twenty-four hours later, embryoid bodies were washed and sown in ultralow adherent plates at 600 embryoid bodies per 10 cm^2^ in CellGroSCGM medium (CellGenix) supplemented with TPO (100 ng ml^−1^, Cellgenix) and SCF (25 ng ml^−1^, Gibco). At day 10, embryoid bodies were dissociated to single cells using Collagenase-IV and Dispase-II (1 mg ml^−1^, Gibco) followed by enzyme free cell dissociation buffer (Gibco) treatment. Single cells were cultivated at 2E+5 per ml on tissue culture plates (Corning) for an additional 10 days in CellgroSCGM with TPO (100 ng ml^−1^) and IL1-β (10 ng ml^−1^, Miltenyi Biotec). Half of culture media was renewed every 3 days.

#### Long-term MK-FOP cultures

From day 10 after dissociation to single cells, the cultures were maintained in CellGroSCGM with low TPO concentration (20 ng ml^−1^) and SCF (50 ng ml^−1^). Culture medium was half-renewed every 3 days and cells split every 7–10 days when reaching concentration of 1–1.5E+6 cells per ml.

#### FOPMK freezing

Cells from day 30–70 cultures were collected and frozen at 0.5–2E+6 cells per ml in IMDM 20% fetal bovine serum (Gibco) and 5% DMSO (Sigma).

#### Adherent cell protocol

Small cell clumps were generated from sub-confluent hPSC cultures using a Collagenase-IV and Dispase-II mix and sown on human fibronectin coated (50 μg ml^−1^, Millipore) wells in CDM-containing FGF2 and Activin-A (15 ng ml^−1^ each) at an approximate density of 2–5E+5 cells per 10 cm^2^. Cells were transduced with LVPs the next day and kept for 2 days in FGF2+Activin-A (pluripotency) or FGF2+LY-294002+BMP4 (ref. 25) (mesoderm) depending on experiment settings. From day 2, cells were maintained in CellgroSCGM with TPO and SCF as described above.

#### MK-directed differentiation

hPSC lines were differentiated as described^27^ using batch tested serum and stromal cells from Prof Koji Eto Laboratory.

### Cord blood-derived megakaryocytes

Cord blood was obtained after informed consent under a protocol approved by the Cambridgeshire 4 Research Ethics Committee (07/MRE05/44). CD34-positive cells (≥98%) isolated by magnetic cell sorting (Myltenyi Biotec) were seeded at 1E+5 cells per ml in CellgroSCGM containing TPO (100 ng ml^−1^) and IL1-β (10 ng ml^−1^) and cultivated for 10 days. We routinely obtained 70–90% CD41a+ and 20–60% CD42a+ cells by the end of the culture.

### Flow cytometry analysis

Flow cytometry experiments were performed on a CyAn ADP (Beckman Coulter). Single-cell suspensions were generated using a Collagenase-IV/Dispase-II mix and/or enzyme free dissociation buffer. Cells were stained for 20 min at room temperature (RT) in PBS 0.5%BSA 2 mM EDTA using combinations of FITC, PE and APC-conjugated antibodies (Supplementary Table 1). Background fluorescence was set against matched isotype control antibodies and compensation matrix defined using single-colour-stained cells. Flow count fluorospheres (Beckman Coulter) and DAPI (1 μg ml^−1^) were used to determine viable cell count in samples.

### Cell morphology and phenotype analysis

#### Cell morphology analysis

Cells were spun onto a glass slide using cytofunnels at 400*g* for 5 min, methanol fixed and stained using the Romanowsky method.

#### Megakaryocyte colony forming assay

Around 5,000 cells per chamber were used in MethoCult methylcellulose assays (#4230, StemCell Technologies) containing screened fetal bovine serum and supplemented with TPO and SCF (100 and 50 ng ml^−1^ respectively); MegaCult collagen cultures (StemCell Technologies) were dehydrated following manufacturer’s instructions for colony immunostaining.

#### Immunofluorescence analysis

Megakaryocytes were cultivated on human fibrinogen (50 μg ml^−1^, Millipore) coated glass cover slips for 48 h to foster adhesion and proplatelet formation. Cells were fixed in 2% formaldehyde, permeabilized with 0.1% Saponin/0.2% Gelatin and incubated 2 h at RT with selected primary antibodies (Supplementary Table 1) then with fluorochrome conjugated secondary antibodies for 45 min atRT. Cell nuclei were stained with DAPI. Images were acquired on a fluorescent microscope Axiovert 40 (Zeiss) or a SP5 confocal microscope for granule imaging (Leica).

#### Ploidy analysis

Cells were fixed using 4% formaldehyde for 10 min at RT, immunostained for CD41a and CD42a expression and subsequently incubated in PBS 0.1% Tween with DAPI at 1 μg ml^−1^ for 15 min at RT before flow cytometry analysis.

#### Transmission electron microscopy

Megakaryocytes were fixed in 2% glutaraldehyde/formaldehyde followed by post fixation, resin embedding and staining as previously described^17^,^64^, and eventually analysed on a FEI Tecnai G2 microscope.

### Gene expression analysis by RT-qPCR

Total RNA was extracted using RNeasy kits (Qiagen) according to the manufacturer’s instructions including DNase treatment. cDNA was prepared from 250–500 ng total RNA using Maxima First Strand cDNA Synthesis Kit (random hexamers and oligo(dT)_18_ mix for priming; Fermentas). Two-step qPCR reactions were performed in duplicates using SYBR green chemistry on ABI 7500HT or Mx3000P instruments (Applied Biosystems; Agilent Technologies). Relative gene expression was calculated by the 2^−Δct^ method using HMBS as housekeeping gene for normalization. qPCR primer pairs (Supplementary Table 2) designed to amplify only cDNA, to detect all known isoforms, and to have no reported off-target matches searching the human NCBI RefSeq database were tested within 80–120% PCR efficiencies with single dissociation curves. We used UTR targeting (absent from transgenes) to monitor endogene expression while transgene specific primer pairs used a common reverse primer specific to the viral vector RNA.

### Whole-genome expression microarray analysis

DNA-free total RNA was extracted as above from sorted CD42b+ cell fractions using the EasySep system (Stemcell Technologies; >95% purity, for cbMK, ddMK and fopMK samples) or unsorted >80% CD42b+ cells (LT-fopMK samples) and 500 ng hybridized to Illumina Human HT-12 v4 BeadArrays.

#### Microarray gene expression

Microarray signal intensities from Genome Studio version1.9 were variance stabilization transformed and robust spline normalized (RSN). For differential analysis, we removed low signal probes with detection *P* value>0.01 in all samples, leaving only informative probes. These procedures were carried out using R package Lumi. For each pairwise differential analysis, we applied surrogate variable analysis (SVA) to correct for un-modelled factors that may bring about batch effects. We then tested for differential expression using limma where statistical significance was set to 2-fold change and 5% false discovery rate or Benjamini-Hochberg adjusted *P* value.

#### Hierarchical clustering

Hierarchical clustering was performed using R package pvclust. For dissimilarity or distance measure, we use 1-correlation and average as agglomerative method.

#### Gene ontology enrichment analysis

We performed enrichment analysis using GOstat 2.24 of over-represented gene ontology terms in the set of differentially expressed genes. We used standard hypergeometric test using only informative probes in the chip as the universe.

#### Gene set enrichment analysis

We used gsea2–2.0.13 with datasets for megakaryocytes (*n*=4) and other blood cells (*n*=46) from the Haematlas study^23^ (E-TABM-633; Illumina Human-6v2 array).

#### Principal component analysis

Classification of samples in multiple dimensional factor spaces was applied by calling the function *cmdscale*.

#### Gene expression heatmaps

Heatmap builder v1.1 (Dr Ashley lab, Stanford) was used for dataset normalized representations.

### *In vitro* platelet analysis

#### Co-culture on feeder cells

To promote platelet production, day 10 cbMKs and day 20–90 fopMKs were further cultivated for 48 h in CellgroSCGM plus Heparin (25 U ml^−1^) without further cytokine addition at 1E+5 cells per cm^2^ on gamma-irradiated C3H10T1/2 feeder cells (Riken Institute; 1E+4 cells per cm^2^).

#### Platelet flow analysis

Crude supernatant containing the platelets was analysed by flow cytometry after addition of 1:9 volume of acid citrate dextrose (ACD, Sigma) and cell removal by centrifugation 150*g* at 10 min. Antibodies against human platelet receptors were added directly to the media (1:50 dilution, see Supplementary Table 1) for 30 min at RT and unwashed platelets analysed using fluorospheres for quantification. Human platelets from fresh whole-blood diluted in PBS/ACD or from day 8–10 platelet concentrate units (NHSBT, UK) were analysed in parallel.

#### Washed platelet preparation

Human platelet-rich plasma and *in vitro* generated platelets collected as above were washed following the optimized protocol from Cazenave *et al*.^65^ to prevent activation and maximize platelet function preservation.

#### Platelet size analysis

Washed platelets were run on a Sysmex XE-2100 Automated Hematology System.

#### Electron microscopy

Washed platelets were fixed and stained as described for MKs.

#### Spreading assay

Washed platelets in Tyrode-HEPES buffer complemented with CaCl_2_ 1 mM were sown on fibronectin coated glass cover slips and incubated 45 min at 37 °C. They were subsequently fixed using 2% formaldehyde, immunostained and analysed as described for MKs.

#### Platelet survival *in vivo*

Washed platelets were injected to macrophage depleted (by clodronate liposome injection at day −3) 8–12-week-old male NOD *scid* gamma (NOD.Cg-Prkdc scid Il2rg tm1Wjl /SzJ; NSG) mice through the tail vein as single dose of 2E+7 CD41a/42a++ platelets. The human versus mouse platelet content was monitored by flow cytometry from whole-blood samples at 1, 30, 120 min and 24 h after transfusion using antibodies specific for human and murine CD41a. The absolute human platelet count was determined using flow count fluorosphere (Beckman Coulter) and platelet survival calculated by the multiple hits method relative to the 30 min equilibrium time point^66^,^67^. All mice were kept in specific pathogen-free conditions, and all procedures were performed according to the United Kingdom Home Office regulations and approved by the University of Cambridge Animal Welfare Ethical Review Body.

#### *In vitro* thrombus formation in laminar flow

Human blood or *in vitro* platelets were stained with Calcein-AM (100 nM; Life Technologies) for 10 min at 37 °C before being washed and defined amount were mixed with 1 ml of human blood collected in 3.2% Citrate. Thrombocytopenic blood was artificially prepared by depleting the platelets from plasma by performing 2 sequential centrifugation steps at 2,200*g* for 10 min and washing the red blood cell fraction with Tyrode-HEPES buffer before blood reconstitution. The procedure for clot formation under flow was modified from de Witt *et al*.^68^ Briefly, glass slides were locally coated with Horm collagen spots (50 μg ml^−1^) and mounted into a flow chamber placed under a fluorescent microscope (EVOS system, Advanced Microscopy Group). The blood was then perfused through the chamber at 1,600 s^−1^ (7.2 ml h^−1^) for 3 min allowing thrombi formation on collagen spots and imaging subsequently performed. Image analysis was carried on using ImageJ recording thrombus area and calcein-AM events per clot.

#### Thrombus formation intravital imaging

Washed platelets were labelled with Calcein-AM and injected to macrophage depleted 8–12-week-old male NSG mice through the carotid artery after cannulation as single dose of 5E+7 CD41a/42a/Calcein-AM+++ platelets. Thrombus formation was induced from 15 min onwards after platelet transfusion by micropoint laser injury of the cremaster vessel walls as previously described^39^. Fluorescent and bright fields were simultaneously recorded by confocal microscopy and analysed using SlideBook 6 (Intelligent Imaging Innovations). The number of Calcein-AM+ human platelets incorporated in mouse thrombi until maximal size was reached was recorded and reported to thrombus area. All animal experiments have been approved by the United Kingdom Home Office and the Birmingham University Animal Welfare Ethical Review Body.

#### Flow cytometry aggregation assay

Modified from original protocol^38^ to include the live–dead particle discriminator Calcein-AM, it allows quantification of platelet aggregation using small numbers of platelets (2E+6 per reaction). Duplicate platelet samples were stained with Calcein-AM and anti-CD31 (clone WM59) conjugated with APC or V450 in HEPES buffer supplemented with 20 μM PPACK dihydrochloride (Calbiochem), washed then mixed in the presence or absence of the agonists thrombin receptor-activating peptide (TRAP) and ADP (10 μM each). Percentage aggregation was determined by flow cytometry for Calcein-AM-positive double CD31-positive events.

#### Bead platelet adhesion flow cytometry assay

Modified from original protocol^36^, it quantifies adhesion of platelets to single 20μM polystyrene beads (Sigma) coated with either BSA or Fibrinogen. 3 × 10^5^ platelets stained with 100 nM Calcein-AM were mixed in basal culture media with either 20 μl BSA or 20 μl fibrinogen-coated beads, agonists (TRAP and ADP as above), incubated for 10’/37 °C and subsequently stained with anti-CD41a-APC antibody. DAPI negative, Calcein-AM and CD41a positive single beads bound by platelets are quantified by flow cytometry.

### Statistics

Results are presented as mean±standard error of the mean (s.e.m.) with *n* representing the number of biological replicates unless otherwise stated. Statistical *P* values were calculated by two-tail Student’s *t*-test unless otherwise stated.

## Additional information

**Accession codes:** Gene expression microarray data are available from the GEO repository under the accession number GSE54822.

**How to cite this article:** Moreau, T. *et al*. Large-scale production of megakaryocytes from human pluripotent stem cells by chemically defined forward programming. *Nat. Commun.* 7:11208 doi: 10.1038/ncomms11208 (2016).

## Supplementary Material

Supplementary Information Supplementary Figures 1-6 and Supplementary Tables 1-2

Supplementary Movie 1 Confocal 3-D rendering of P-selectin granular staining. Proplatelet forming fopMKs were stained for P-Selectin (Alexa-568/red) and Alpha-Tubulin (Alexa 488/green) and analysed by confocal microscopy followed by 3-dimensional reconstruction of cell volume (Movie S1) and proplatelet protrusion (Movie S2).

Supplementary Movie 2 Confocal 3-D rendering of P-selectin granular staining. Proplatelet forming fopMKs were stained for P-Selectin (Alexa-568/red) and Alpha-Tubulin (Alexa-488/green) and analysed by confocal microscopy followed by 3-dimensional reconstruction of cell volume (Movie S1) and proplatelet protrusion (Movie S2).

Supplementary Movie 3 Live monitoring of thrombus formation in vitro under arterial shear stress. Representative thrombus formation (10-fold normal speed; Movie S3). Visualisation of Calcein-AM labelled spiked platelets during thrombus formation (normal speed; Movie S4).

Supplementary Movie 4 Live monitoring of thrombus formation in vitro under arterial shear stress. Representative thrombus formation (10-fold normal speed; Movie S3). Visualisation of Calcein-AM labelled spiked platelets during thrombus formation (normal speed; Movie S4).

Supplementary Movie 5 Real-time intravital microscopy of thrombus formation in vivo. Visualisation of human Calcein-AM labelled transfused platelets behaviour - donor platelets (Movie S5) and fopMK platelets (Movie S6) - during thrombus formation (5-fold normal speed).

Supplementary Movie 6 Real-time intravital microscopy of thrombus formation in vivo. Visualisation of human Calcein-AM labelled transfused platelets behaviour - donor platelets (Movie S5) and fopMK platelets (Movie S6) - during thrombus formation (5-fold normal speed).
