# Corrigendum: Large-scale production of megakaryocytes from human pluripotent stem cells by chemically defined forward programming

**DOI:** 10.1038/ncomms15076

**Published:** 2017-07-28

**Authors:** Thomas Moreau, Amanda L. Evans, Louella Vasquez, Marloes R. Tijssen, Ying Yan, Matthew W. Trotter, Daniel Howard, Maria Colzani, Meera Arumugam, Wing Han Wu, Amanda Dalby, Riina Lampela, Guenaelle Bouet, Catherine M. Hobbs, Dean C. Pask, Holly Payne, Tatyana Ponomaryov, Alexander Brill, Nicole Soranzo, Willem H. Ouwehand, Roger A. Pedersen, Cedric Ghevaert

Nature Communications 7 Article number: 11208 ; DOI: 10.1038/ncomms11208 (2016) Published 04 07 2016; Updated 07 28 2017

This Article contains an error in the author contributions section that has resulted in incorrect credit for preparation and critical analysis of the manuscript. The correct author contributions section is as follows:

‘T.M. designed, analysed, performed and interpreted most experiments and wrote the paper. A.L.E. performed and analysed experiments. L.V. and Y.Y. performed and interpreted bioinformatics analyses pertaining to the genomic characterization of the MKs. M.R.T. contributed to megakaryocyte characterization and wrote the paper. M.W.T. performed and interpreted bioinformatics analyses pertaining to the forward programming concept. M.C. and M.A. contributed to megakaryocyte characterization. D.H., W.H.W., C.M.H., A.D., R.L., G.B. and D.C.P. performed experiments. H.P., T.P. and A.B. designed and performed intravital experiments and analysed data, A.B. also participated in preparation and critical analysis of the manuscript. N.S. interpreted bioinformatics analyses pertaining to the genomic characterization of the MKs. W.H.O. contributed platelet expert input to the forward programming concept. R.A.P. conceived the forward programming approach, designed, analysed and interpreted experiments and wrote the paper. C.G. drove the platelet biology, designed, analysed and interpreted experiments and wrote the paper.’

