# Supplementary material for: Large-scale production of megakaryocytes from human pluripotent stem cells by chemically defined forward programming
Source: Nat Commun. 2016 Apr 7;7:11208. doi: 10.1038/ncomms11208 (PMC4829662; doi:10.1038/ncomms11208)
Supplement: Supplementary Information — Supplementary Figures 1-6 and Supplementary Tables 1-2 [file ncomms11208-s1.pdf]

a

CB-MK vs. hESC comparative analysis					
Expression RANK	Entrez_Gene ID	Symbol	log2(MK-ESC)	Internal Nodes	Chromatin nodes
1	2313	FLI1	6.218082146	2	0
2	4176	MCM7	5.986352868	1	1
3	2623	GATA1	4.962946076	6	5
4	4208	MEF2C	4.900404526	1	6
5	8328	GFI1B	4.495031127	0	0
6	6886	TAL1	3.985331451	1	3
7	861	RUNX1	3.902	0	5
8	29883	CNOT7	3.694630861	0	0
9	10538	BATF	2.981322104	0	0
10	10320	IKZF1	2.924327784	2	6
11	54816	ZNF280D	2.904703907	0	0
12	7704	ZBTB16	2.842398404	1	9
13	6915	TBXA2R	2.807151272	0	0
14	6688	SPI1	2.545218071	2	5
15	56978	PRDM8	2.421821704	0	0
16	2122	MECOM	2.413061623	0	3
17	10661	KLF1	2.377123936	1	1
18	25946	ZNF385	2.35559522	0	1
19	4149	MAX	2.232674581	0	2
20	79230	ZNF557	2.227154539	0	0
21	864	RUNX3	2.142642552	0	6
22	10194	TSHZ1	2.112852011	0	0
23	168544	ZNF467	2.087691342	1	0
24	51157	ZNF580	2.049279855	0	0
25	51341	ZBTB7A	2.040210037	0	4
26	90594	ZNF439	2.031431266	0	0
27	3202	HOXA5	1.919975036	3	1
28	23414	ZFPM2	1.907889051	1	1
29	7128	TNFAIP3	1.852543925	0	0
30	79192	IRX1	1.832799737	0	0
31	4772	NFATC1	1.806170804	1	2
32	6775	STAT4	1.741736537	1	1
33	5970	RELA	1.71499009	1	10
34	146542	ZNF688	1.642303935	0	0
35	7507	XPA	1.556867603	0	1
36	3229	HOXC13	1.546458879	0	0
37	4782	NFIC	1.529451132	0	1
38	121274	ZNF641	1.507346451	0	0
39	79175	ZNF343	1.479242387	0	0
40	55897	MESP1	1.464070356	0	0
41	3212	HOXB2	1.343797629	0	2
42	22806	ZNFN1A3	1.329858707	1	4
43	4097	MAFG	1.292335842	0	1
44	6304	SATB1	1.264860686	0	3
45	2306	FOXD2	1.228418006	0	0
46	6915	TBXA2R	1.200562225	0	0
47	7490	WT1	1.185142601	0	1
48	58508	MLL3	1.169828436	2	0
49	5325	PLAGL1	1.164040819	0	3
50	2355	FOSL2	1.10592127	0	0
51	25913	POT1	1.096700285	0	0
52	6945	MLX	1.078171011	0	0
53	163059	ZNF433	1.07087317	0	0
54	54921	DERPC	1.0671444	0	0
55	1388	CREBL1	1.056454596	0	0
56	4297	MLL	1.052634651	2	3
57	3297	HSF1	1.050663495	0	4
58	79027	ZNF655	1.036140954	0	0
59	54617	INOC1	1.028943464	0	0
60	92822	ZNF276	1.025727446	0	0
61	79724	FLJ23436	0.954750193	0	0
62	1389	CREBL2	0.930665715	0	0
63	1745	DLX1	0.907017189	0	0
64	50804	MYEF2	0.898223319	0	0
65	9575	CLOCK	0.892683013	0	2
66	5252	PHF1	0.873134407	5	0
67	9191	DEDD	0.854743494	0	0
68	54862	CC2D1A	0.84994884	0	0
69	10847	SRCAP	0.823139145	0	2
70	7290	HIRA	0.80510379	2	-
71	267004	PGBD3	0.778657087	0	0
72	163087	ZNF383	0.75853371	0	0
73	4205	MEF2A	0.742000993	1	5
74	3203	HOXA6	0.720932756	0	0
75	221785	ZNF498	0.719656867	0	0
76	388569	FLJ45850	0.716863475	0	0
77	122953	JDP2	0.69730326	0	2
78	7629	ZNF76	0.681751808	0	2
79	7067	THRA	0.670073694	1	5
80	3508	IGHMBP2	0.634230623	0	0
81	2145	EZH1	0.627585534	0	0
82	7067	THRA	0.626375578	0	4
83	64763	ZNF574	0.534902684	0	0
84	64412	ZNF336	0.508063874	0	0
85	339559	ZNF642	0.489980158	0	0
86	4221	MEN1	0.488010277	2	2
87	57684	ZBTB26	0.486487546	0	0
88	4798	NFRKB	0.482938158	0	0
89	27343	POLL	0.477708994	0	0
90	54885	FLJ20298	0.469046252	0	0
91	3642	INSM1	0.461211931	0	2
92	79973	ZNF442	0.379657793	0	0
93	84330	ZNF414	0.367386704	0	0
94	7727	ZNF174	0.265735517	0	0
95	54993	ZSCAN2	0.078694232	0	0
-	4211	MEIS1	-	3	1
-	5087	PBX1	-	2	0
-	1831	TSC22D3	-	1	2
-	4778	NFE2	-	1	1
-	161882	ZFPM1	-	1	0
-	55544	RBM38	-	0	0

101 DNA binding protein candidates

Removal of 21 histone genes from 116 MK specific identified DNA binding genes
Addition of MEIS1, NFE2,PBX1,ZFPM1,RBM38,TCSD to the list
(literature pick, blue highlight)

b

Candidate ranking				
Candidate RANK	Symbol	log2(MK-ESC)	Internal Nodes	Chromatin nodes
1	**GATA1**	4.962946076	6	5
2	IKZF1	2.924327784	2	6
3	**SPI1**	2.545218071	2	5
4	HOXA5	1.919975036	2	1
5	**MEIS1**	-	2	1
6	**FLI1**	6.218082146	2	0
7	**PBX1**	-	2	0
8	**ZBTB16**	2.842398404	1	9
9	ZNFN1A3	1.329858707	1	4
10	**TAL1**	3.985331451	1	3
11	NFATC1	1.806170804	1	2
12	TSC22D3	-	1	2
13	KLF1	2.377123936	1	1
14	ZFPM2	1.907889051	1	1
15	STAT4	1.741736537	1	1
16	**NFE2**	-	1	1
17	ZNF467	2.087691342	1	0
18	ZFPM1	-	1	0
19	RELA	1.71499009	0	10
20	**MEF2C**	4.900404526	0	6
21	RUNX3	2.142642552	0	6
22	RUNX1	3.902	0	5
23	ZBTB7A	2.040210037	0	4
24	HSF1	1.050663495	0	4
25	MECOM	2.413061623	0	3
26	SATB1	1.264860686	0	3
27	PLAGL1	1.164040819	0	3
28	MAX	2.232674581	0	2
29	HOXB2	1.343797629	0	2
30	CLOCK	0.892683013	0	2
31	ZNF385	2.35559522	0	1
32	XPA	1.556867603	0	1
33	NFIC	1.529451132	0	1
34	MAFG	1.292335842	0	1
35	WT1	1.185142601	0	1
36	GFI1B	4.495031127	0	0
37	CNOT7	3.694630861	0	0
38	BATF	2.981322104	0	0
39	ZNF280D	2.904703907	0	0
40	TBXA2R	2.807151272	0	0
41	PRDM8	2.421821704	0	0
42	ZNF557	2.227154539	0	0
43	TSHZ1	2.112852011	0	0
44	ZNF580	2.049279855	0	0
45	ZNF439	2.031431266	0	0
46	RBM38	-	0	0

46 Transcription Factor candidates

Analysis of documented protein-protein interactions (VisANT)
Sorting on:
1.number of internal interactions
2.number of interactions with chromatin remodelling factors
3.differential gene expression
4. exclusion of low DE genes (log2 <1)
5. exclusion of zero nodes low DE genes (log2 <2)
6. Atypic exclusion of MLL, MLL3 (technical, cloning in lentiviruses not possible); MCM7 (mini chromosome maintenance)
7. Atypic inclusion of CLOCK (HAC activity)

c

Gene list for epigenome modifiers		
HDAC	HAC	DNMT
HDAC1	CREBBP	DNMT1
HDAC2	CDY1	DNMT3A
HDAC3	CDY2	DNMT3B
HDAC4	CDYL1	
HDAC5	CLOCK	
HDAC6	ELP3	
HDAC7A	EP300	
HDAC8	HAT1	
HDAC9	KAT2A	
HDAC10	KAT2B	
HDAC11	KAT5	
SIRT1	MYST1	
SIRT2	MYST2	
SIRT3	MYST3	
SIRT4	MYST4	
SIRT5	NCOA1	
SIRT6	NCOA3	
SIRT7	NCOAT	
	TF3C4	

Chromatin remodelling factors and DNA methyl transferases used for the chromatin remodelling factor interaction analysis

Supplementary Figure 1. Selection process for MK-FOP transcription factor candidates. (**a**) List of the 101 DNA protein binding genes up-regulated in cbMKs vs. the H9 hESC line (see *online materials* for details). Twenty one

histone coding genes have been excluded and 6 additional TFs added based on literature (highlighted in blue). Log2 values of differential expression, number of internal/epigenome modifier interactions (reported from VisANT) are indicated (**b**) Final 46 TF candidates ranked list. The 9 TFs used in this study are highlighted in grey. (**c**) Epigenome modifier coding genes used for interaction screening. HDAC: Histone Deacetylases; HAC: Histone acetyltransferases; DNMT: DNA Methyltransferases.

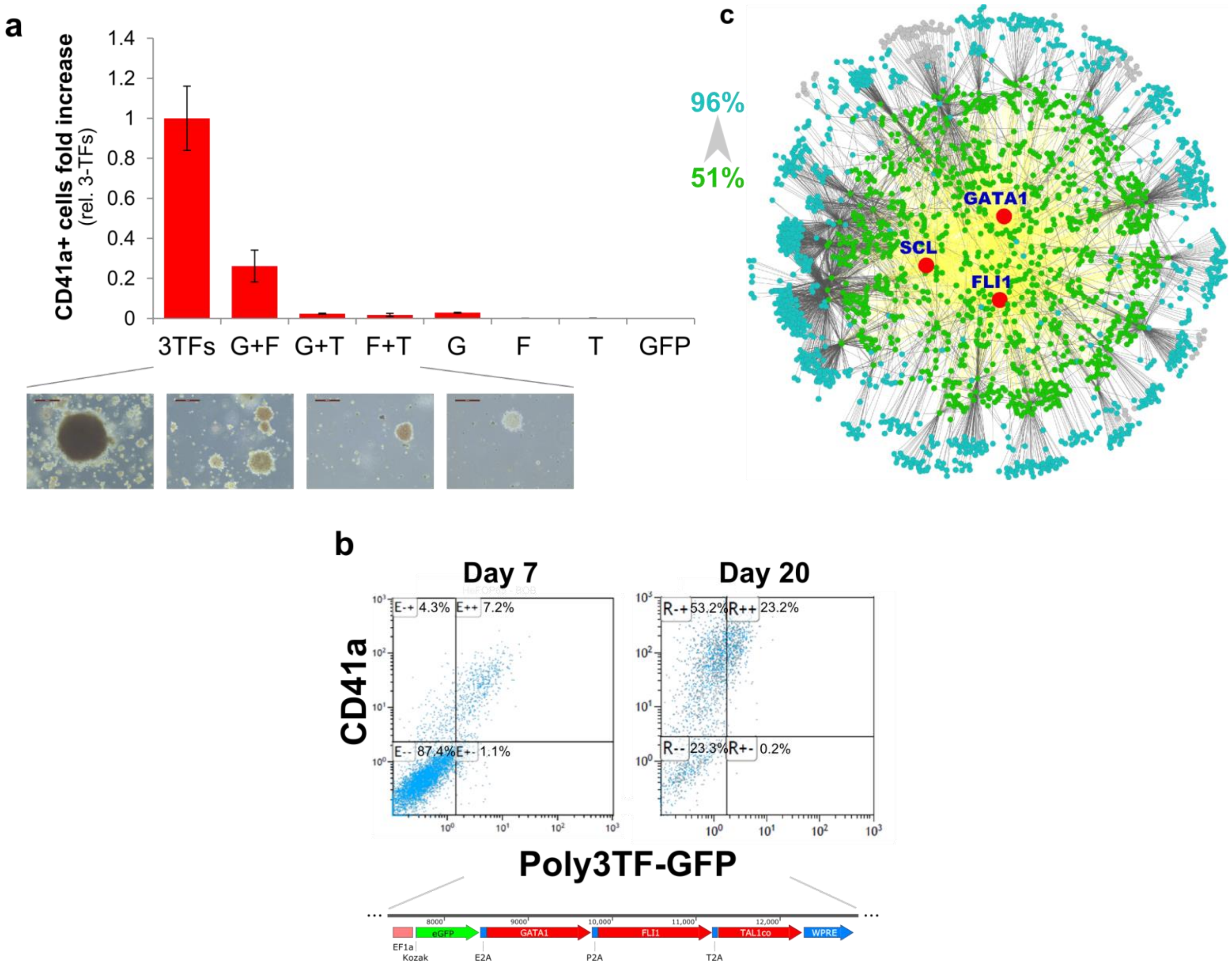

Supplementary Figure 2. Combined expression of the 3-TFs induces MKFOP. (**a**) Representative microscopy fields from clonogenic assays initiated from day 7 cultures transduced with different combinations of the 3-TFs (hiPSC#1; 3-TFs, G+F, G+T, F+T respectively) showing higher colony density and larger colonies for the combined 3TFs than any other combination. (**b**) Combined CD41a and GFP expression detected by flow cytometry from day 7 and day 20 cells transduced with a single polycistronic vector coding for the 3-TFs and the GFP reporter gene (2A peptide vector; hiPSC#1). (**c**) Annotated Cytoscape MK gene network (Gieger et al.,2011; 2,125 nodes) showing intersection with 3-TFs ChIP-seq data (Tijssen et al., 2011). The regulatory regions of 51% of the MK genes are bound by at least one of the 3-TFs (green nodes) which are further linked to 96% of the entire network through first degree interactions (blue nodes).

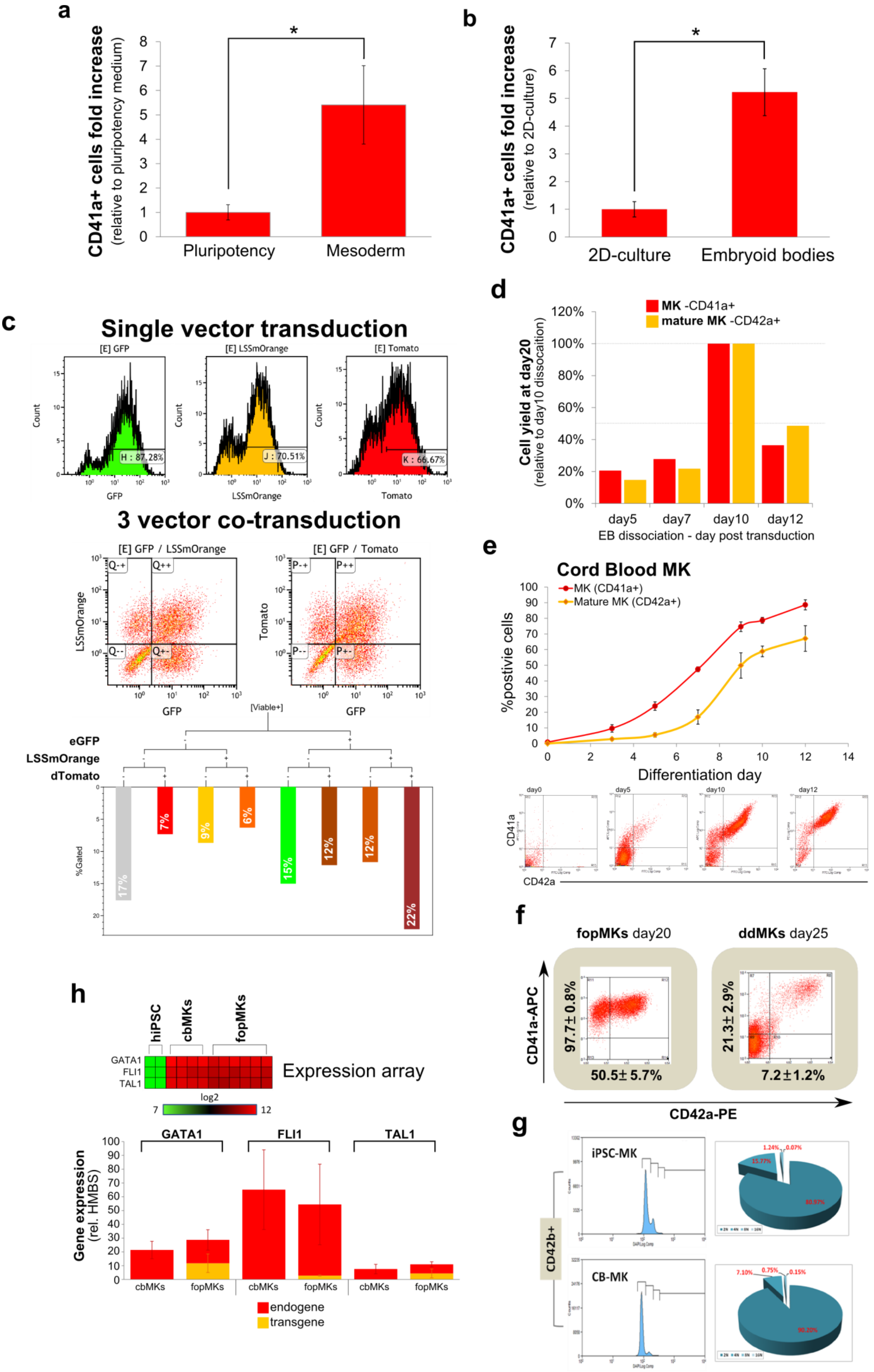

Supplementary Figure 3. Optimisation of a chemically-defined MKFOP protocol. (**a**) The hiPSC#1-2 lines sown as clumps on fibronectin-coated plates were transduced by the 3-TFs and maintained in pluripotency (FGF2+activinA) or mesoderm-inducing (FGF2+BMP4+LY-294002) medium for 2 days followed by MK medium (TPO+SCF) for a further

5 days. The percentage of CD41a+ cells was measured by flow cytometry on day 7. Bar graphs show the fold increase of CD41a+ cells relative to pluripotency medium (mean ±sem; n=4, **P*<0.05 by 2-tail T-test). (**b**) The hiPSC#2 line was sown as clumps or single cells on fibronectin- or gelatine-coated plates (collectively 2-D culture), or sown as embryoid bodies induced by forced aggregation of single cells (Embryoid bodies, EB) concomitantly to transduction with the 3-TFs. Cells were then maintained for 2 days in mesoderm-inducing medium followed by 5 days in MK medium. Bar graphs represent the CD41a+ cell-fold increase relative to 2D-culture (mean ±sem, n=4, 3 respectively, **P*<0.05 by 2-tail T-test). (**c**) the hiPSC#1 was transduced with separate pWPT lentiviral vectors coding for the reporter proteins eGFP, dTomato or LSSmOrange. The percentages of cells (co-)expressing each reporter after transduction at MOI20 by single vectors (top histograms) or co-transduction with three vectors (middle dot plots and bottom bar graph) are indicated. (**d**) The impact of EB dissociation timing on day 20 FOPMKs output was tested from day 5 to 12. The MK and mature MK yield (cell count at day 20) is shown relative to day 10 dissociation (hiPSC#1). (**e**) Time course analysis of cbMK differentiation including line plots and representative flow cytometry dot plots for CD41a and CD42a expression (mean ±sem; n=3). (**f**) Representative flow cytometry dot plots from MK-FOP culture at day 20 and MK-DD at day 25 (mean ±sem; n=4, 3 respectively, hiPSC#1). (**g**) Ploidy analysis of CD42b+ fopMKs day 20 and cbMKs day 10 by flow cytometry. Representative histograms and percentages of 2, 4, 8 and 16N cells are shown (mean; n=3 and 2 respectively). (**h**) The expression levels of the 3-TFs in cbMKs (day10) and fopMKs (day20) are shown from the whole genome expression array data (top row normalized heatmap) and from RT-qPCR analysis (bottom bar graph; mean ±sem; n=3/3 for cbMKs and fopMKs (hiPSC#1, #3 and #4) respectively). The latter discriminates endogenous from transgenic expression using specific primers described in Methods and Supplementary Table2.

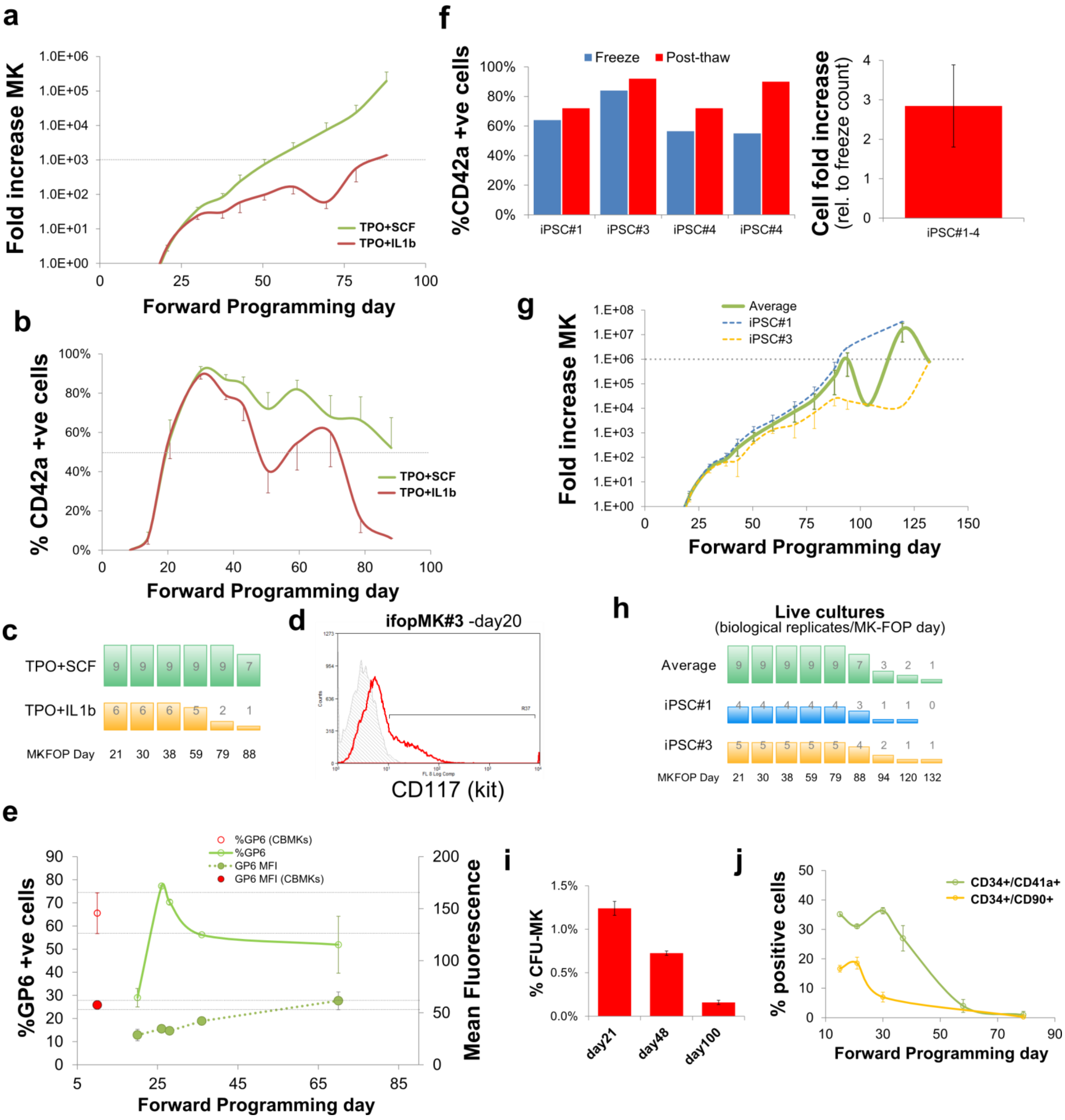

Supplementary Figure 4. Long-term MKFOP culture properties. (**a-c**) The cumulative mature MK-fold increase relative to day 0 hiPSC cell input, percentages of CD42a+ cells and culture survival (biological replicate alive at a given time point) are shown for TPO+SCF vs. TPO+IL1β culture conditions (mean ±sem, hiPSC lines #1 and #3). (**d**) Expression of CD117 (KIT, SCF receptor) in MK-FOP culture. (**e**) The percentage of GP6 expressing cells and GP6 staining mean fluorescence intensity are shown over time for LT-fopMK culture (mean ±sd, hiPSC lines #1 and #3, n=2) and CBMKs at day 10 (mean±sem, n=7). (**f**) LT-fopMK recovery after freezing. The percentages of CD42a+ cells on the day of freezing (day 30-40 of MK-FOP) and 14 days after thawing are shown for different iPSC lines. The mean ±sem cell fold increase 14 days after thawing is shown for the corresponding lines. (**g-h**) Cumulative MK expansion and biological replicate survival at extended time points (>day 90). (**i**) Clonogenic potential from whole MK-FOP cultures was tested at different time points. The percentage of MK colony forming cells is shown (mean ±sd; hiPSC#1, n=2). (**j**) Percentages of MK progenitors (CD41a+ and CD34+) and hematopoietic progenitors (CD90+ and CD34+) were monitored by flow cytometry through long term MK-FOP cultures (mean ±sd; hiPSC#1 and #3, n=3).

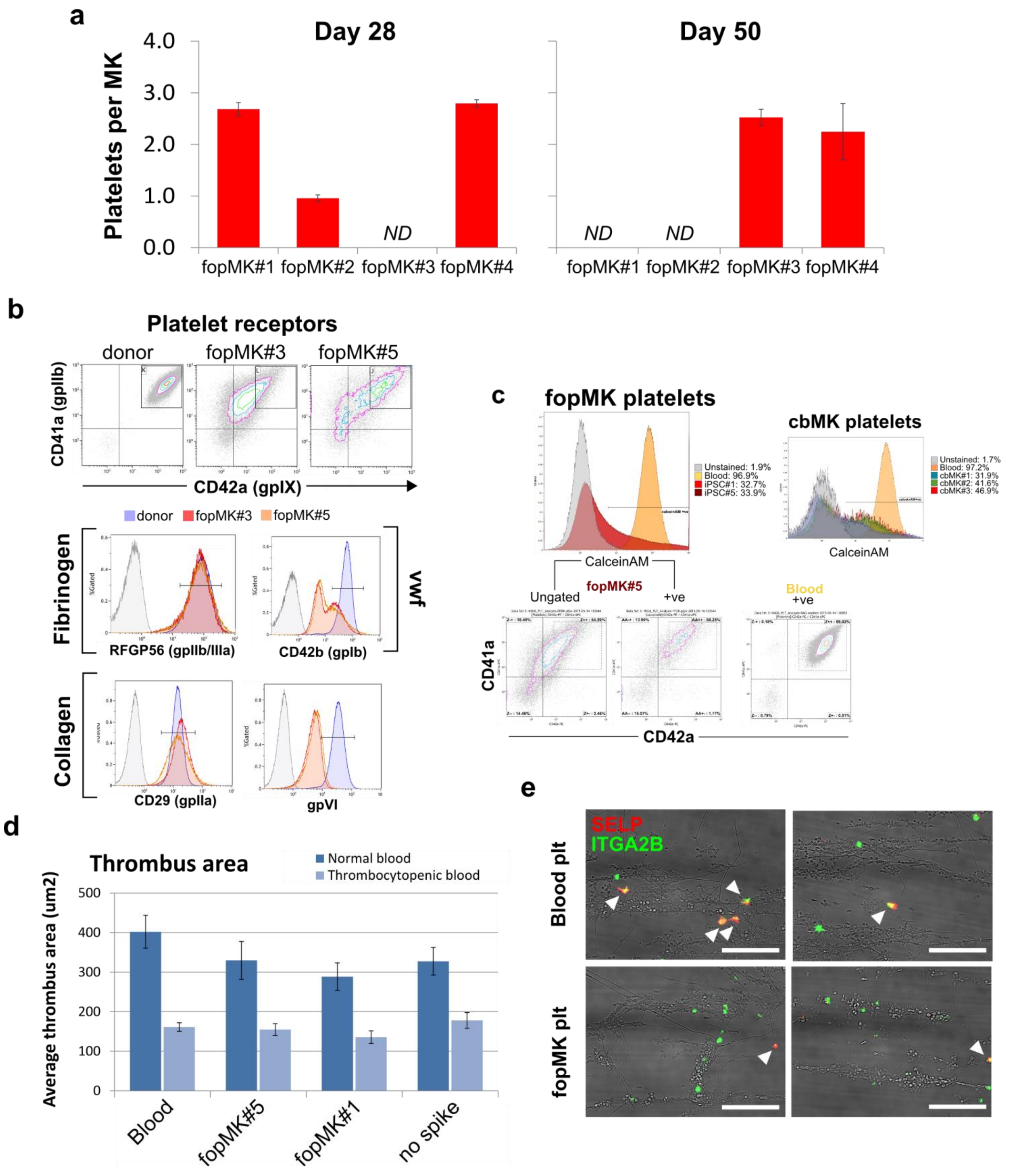

Supplementary Figure 5. ***In vitro*** **platelet release from fopMKs.** (**a**) Average platelet generation per MK in co-culture with C3H10T1/2 feeder cells at different culture time points depending on the hiPSC line forward programmed (mean ±sd; hiPSC#1-4; day 28: n=6, 2, nd, 3; day 50: n=nd, nd, 4, 2 respectively, nd=not done). (**b**) Expression of platelet receptors on washed donor and *in vitro* fopMK platelets. Primary staining for CD41a and CD42a expression (top dot plots) was used for gating on highly expressing platelets similar to blood platelets (black box) for further platelet receptor expression analysis (bottom histograms). (**c**) Representative staining by Calcein-AM of *in vitro* fopMK and donor platelets. The corresponding CD41a/CD42a phenotype detected by flow cytometry with or without gating on Calcein-AM positive events is shown below. (**d**) Average ±sem of individual thrombus area from *in vitro* thrombus assay under shear stress for normal and thrombocytopenic blood. (**e**) Representative pictures from *in vitro* thrombus formation assays. Washed platelets from human blood or fopMKs were mixed into mouse blood ($5x10^7$/ml) and thrombus formation on collagen-coated slides under arterial shear flow monitored by real time microscopy. Human platelets were subsequently detected by immunostaining of human CD41a (ITGA2B) and P-selectin (SELP). Arrowheads point to activated platelets exposing P-selectin at their surface following degranulation. Scale bars 50μm.

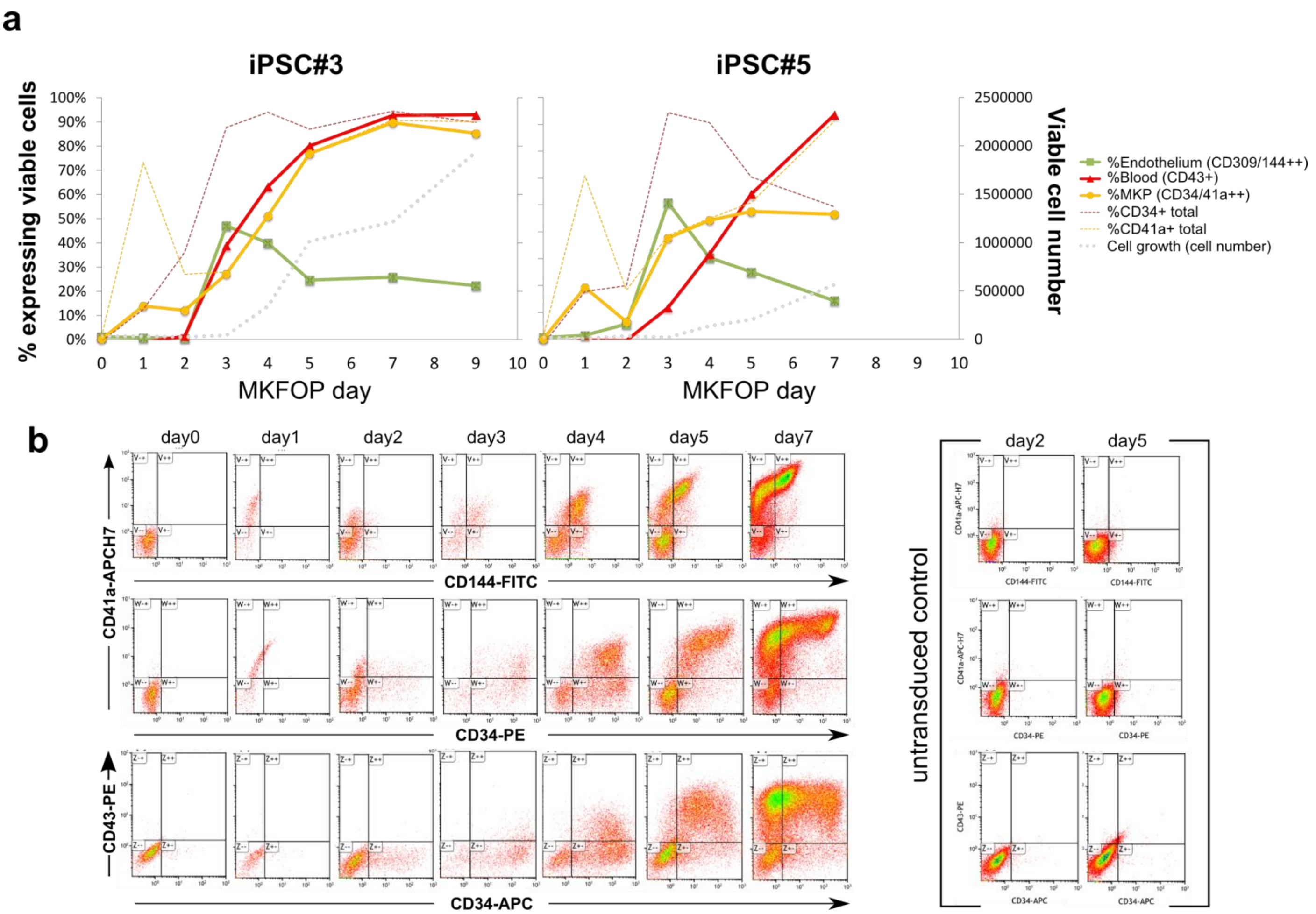

Supplementary Figure 6. ***MK-FOP time course flow cytometry analysis for hemogenic endothelium and blood markers*.** (**a**) the percentage of cells expressing endothelial (CD34, CD144/VE-Cadherin, CD309/FLK1) and blood markers (CD43, CD41a) are shown for hiPSC#3 and #5 from day 0 to day 7-9 post 3-TF transduction. The total viable cell count is indicated as a grey dotted line plotted on the secondary axis. (**b**) Flow cytometry dot plots from hiPSC#5 MK-FOP time course are shown, including phenotype of non-transduced control cells at day 2 and 5.

Supplementary Table 1. Antibodies used for flow cytometry analyses and immunostaining for microscopy studies.

Antigen	Antibody	Fluorochrome	Assay concentration	Manufacturer
CD41a	HIP8	FITC	1:10 dilution (stock)	BD Pharmingen
CD41a	HIP8	PE	1:10 dilution (stock)	BD Pharmingen
CD41a	HIP8	APC	1:10 dilution (stock)	BD Pharmingen
CD42a	ALMA.16	FITC	1:10 dilution (stock)	BD Pharmingen
CD42a	ALMA.16	PE	1:10 dilution (stock)	BD Pharmingen
CD42b	HIP1	PE	1:10 dilution (stock)	BD Pharmingen
CD61	VI-PL2	FITC	1:10 dilution (stock)	BD Pharmingen
GPVI	HY101	PE	2μg/ml	NHSBT Bristol
CD41a/61 complex	RFGP56	APC	2μg/ml	NHSBT Bristol
CD31	WM59	APC	1μg/ml	eBiosciences
CD31	WM59	V-450	1μg/ml	eBiosciences
vWF	Polyclonal	-	10μg/ml	Millipore
Thrombospondin	A6.1	-	1μg/ml	Abcam
Fibrinogen	-	Alexa-488	50μg/ml	Molecular Probes
P-selectin	Thromb/6	PE	2μg/ml	NHSBT Bristol
P-selectin	H-150	-	2μg/ml	Santa cruz Biotech
α-Tubulin	DM1A	-	1:500 dilution (stock)	Sigma Aldrich
F-actin	(Phalloidin)	Alexa-488	400nM	Sigma Aldrich
mIgG(H+L)	*secondary*	Alexa-488	2μg/ml	Molecular Probes
mIgG(H+L)	*secondary*	Alexa-568	2μg/ml	Molecular Probes
rIgG(H+L)	*secondary*	Alexa-568	2μg/ml	Molecular Probes
CD41a (mouse)	MWReg30	FITC	30μg/ml	BD Pharmingen

Supplementary Table 2. Oligonucleotides used for RT-qPCR analyses.

Target gene	Primer	Sequence
**GATA1**	Fo	TTGCCACATCCCCAAGGCGG
	Re	GGGGGAGGGGCTCTGAGGTC
**FLI1**	Fo	GGGCTCGGCTGCAGACTTGG
	Re	AGATGGGCTGCCGCTCCGTA
**TAL1**	Fo	AGCAAAGACCCGGGTGTGCATC
	Re	CCTCTAGCTGGGGGTCACTGCG
**ZFPM1**	Fo	GCTCCCTGGAGATCCACATGCG
	Re	ACAGGCAGATCAGGCACACGA
**NFE2**	Fo	GGTGACCCCTGATGTTGCCCTAG
	Re	GGTTCCCGGAAAGCCCAGATGG
**RUNX1**	Fo	GCCAGCGGCATGACAACCCT
	Re	TAGTGCATGCGGGGGTCGGA
**MEIS1**	Fo	AACCAATCGCAAAGCAAGGGGGA
	Re	ATTGGACCACCCCGGGCTACAT
**MEF2C**	Fo	AGACCCGGTAATGGGTGGCCA
	Re	ACGCCAATGATATGCCTGCAGGT
**MPL**	Fo	TGGTGACCGCTCTGCATCTA
	Re	GCAGGAAACTGCCACCTCA
**CXCR4**	Fo	GCCCTCCTGCTGACTATTCC
	Re	GGCAGGATAAGGCCAACCAT
**VWF**	Fo	CAACACCTGCATTTGCCGAA
	Re	TGACCTGTGACAAGGCACTC
**SELL**	Fo	GCGGTGGCTTCTACGATAGG
	Re	CCTAGGTGGCTGTGAGGATTC
**PF4**	Fo	CCGAGTTTCCCATCGCACT
	Re	TGGGAGGTGGTCTTCACACA
**GP6**	Fo	CGGTAGCAGAATTCTCAGAAGC
	Re	GTCTGACTCCTTTGGACTGGC
**FLK1**	Fo	TTTTTGCCCTTGTTCTGTCC
	Re	TCATTGTTCCCAGCATTTCA
**NANOG**	Fo	CATGAGTGTGGATCCAGCTTG
	Re	CCTGAATAAGCAGATCCATGG
**OCT4**	Fo	AGTGAGAGGCAACCTGGAGA
	Re	ACACTCGGACCACATCCTTC
**HMBS**	Fo	GGGAACCAGCTCCCTGCGAAG
	Re	AGCTGTTGCCAGGATGATGGCAC
**MDH1**	Fo	GGGTGTCCTGGACGGTGTCCT
	Re	CCCTTCTTGGCATGGAGCCCAC
**GATA1-TG**	Fo	GGTGGCTCCGCTCAGCTCAT
	Re	GCAGCGTATCCACATAGCGTAAAAGG
**FLI1-TG**	Fo	CCCGCCATCCTAACACCCACG
	Re	GCAGCGTATCCACATAGCGTAAAAGG
**TAL1-TG**	Fo	AGGCGGTGGACTTGAACTTT
	Re	TCTAGCCAGGCACAATCAGC
**MEIS1-TG**	Fo	ATGGGCATGGAGGGGCAGTG
	Re	GCAGCGTATCCACATAGCGTAAAAGG
**MEF2C-TG**	Fo	CGTACGACGGGAGCGACCGA
	Re	GCAGCGTATCCACATAGCGTAAAAGG
**NFE2-TG**	Fo	CCCCGGGGGACCAAGATGGA
	Re	GCAGCGTATCCACATAGCGTAAAAGG
**PBX1-TG**	Fo	ACAGAAGGCCCTGGCAGTGTT
	Re	GCAGCGTATCCACATAGCGTAAAAGG
**SPI1-TG**	Fo	CGCTGCGCAACTACGGCAAGA
	Re	GCAGCGTATCCACATAGCGTAAAAGG
**ZBTB16-TG**	Fo	CCCGCCCGACTGGAGGATAGA
	Re	GCAGCGTATCCACATAGCGTAAAAGG
